# Normal milk microbiome is reestablished following experimental infection with *Escherichia coli* independent of intramammary antibiotic treatment with a third-generation cephalosporin in bovines

**DOI:** 10.1186/s40168-017-0291-5

**Published:** 2017-07-12

**Authors:** Erika K. Ganda, Natalia Gaeta, Anja Sipka, Brianna Pomeroy, Georgios Oikonomou, Ynte H. Schukken, Rodrigo C. Bicalho

**Affiliations:** 1 Department of Population Medicine and Diagnostic Sciences, College of Veterinary Medicine, Cornell University, Ithaca, NY USA; 2 Department of Internal Medicine, School of Veterinary Medicine and Animal Science, University of São Paulo, São Paulo, Brazil; 3 Epidemiology and Population Health, Institute of Infection and Global Health, University of Liverpool, Liverpool, UK; 4 Department of Animal Sciences, Wageningen University, Wageningen, The Netherlands; 5 GD Animal Health, Deventer, The Netherlands

**Keywords:** Milk microbiome, Mastitis, *E. coli*, Ceftiofur, Dairy cattle, Antimicrobial treatment, Milk, Third-generation cephalosporin, Cephalosporins

## Abstract

**Background:**

The use of antimicrobials in food animals and the emergence of antimicrobial resistance are global concerns. Ceftiofur is the only third-generation cephalosporin labeled for veterinary use in the USA, and it is the drug of choice in the majority of dairy farms for the treatment of mastitis. Here, we use next-generation sequencing to describe longitudinal changes that occur in the milk microbiome before, during, and after infection and treatment with ceftiofur. Twelve animals were intramammary challenged with *Escherichia coli* in one quarter and randomly allocated to receive intramammary treatment with ceftiofur (5d) or untreated controls. Serial samples were collected from −72 to 216 h relative to challenge from the challenged quarter, an ipsilateral quarter assigned to the same treatment group, and from a third quarter that did not undergo intervention.

**Results:**

Infection with *E. coli* dramatically impacted microbial diversity. Ceftiofur significantly decreased LogCFUs but had no significant effect on the milk microbiome, rate of pathogen clearance, or somatic cell count. At the end of the study, the microbial profile of infected quarters was indistinguishable from pre-challenge samples in both treated and untreated animals. Intramammary infusion with ceftiofur did not alter the healthy milk (i.e., milk devoid of clots or serous appearance and collected from a mammary gland that shows no clinical signs of mastitis) microbiome.

**Conclusions:**

Our results indicate that the mammary gland harbors a resilient microbiome, capable of reestablishing itself after experimental infection with *E. coli* independent of antimicrobial treatment.

**Electronic supplementary material:**

The online version of this article (doi:10.1186/s40168-017-0291-5) contains supplementary material, which is available to authorized users.

## Background

Mastitis is a prevalent, costly [1, 2] disease in dairy cows that is defined by an increase in milk somatic cell count (**SCC**) as a result of inflammation in the mammary gland, leading to abnormal milk and varying degrees of clinical severity. This condition affects almost 25% of the 9.3 million dairy cows present in the USA every year [3] and negatively impacts animal welfare [4–6] and productivity [7–9]. Recent studies have reported that approximately 80% of all antimicrobials used on American dairy farms are for the treatment or prevention of mastitis [10]. Prevention measures, improved management, and sanitation have reduced the number of contagious mastitis cases and have led to a change in the etiology of the disease in the last decade [3, 11, 12], making opportunistic environmental pathogens, including coliforms, major contributors to clinical mastitis.

Antimicrobial resistance is a global concern and has led to increasing attention regarding the judicious use of antibiotics. Although conflicting evidence is available on whether human, companion, and/or livestock medicine is responsible for the emergence of antimicrobial resistance [13, 14], the livestock industry has been recognized as one of the main causes [13, 15], perhaps due to the amount of antimicrobials used in this sector [16], affecting humans either through direct contact or through the food chain [17, 18]. The increasing demand for animal protein is believed to be accompanied by a significant growth in need for antimicrobial use in food animal production, which is predicted to rise by approximately 67% between 2010 and 2030 [17]. Responsible use of antimicrobials in food animals is paramount for maintenance of both animal and human health [19] and has been one of the policies proposed by the One Health Initiative [20–22]. Ceftiofur is the only third-generation cephalosporin labeled for veterinary use in the USA [3], is considered a critically important antimicrobial for human medicine [23], and is the drug of choice in the majority of dairy operations for intramammary treatment of mastitis [3]. To this date, the effectiveness of the treatment of coliform mastitis has been discussed in an ambiguous manner [24, 25]. Nevertheless, all information regarding the effectiveness of antimicrobial therapy reported so far is based on clinical cure and the ability to isolate and culture a specific pathogen from a mastitic milk sample in a laboratory setting. Culture-independent studies resulted in significant proof for the existence of a resident microbiota in milk in humans [26, 27], bovines [28–30], and other species [31]. In light of constantly advancing molecular techniques, the use of next-generation sequencing led to a paradigm shift in the understanding of the dynamics of health and disease [32, 33] and offers an opportunity to provide evidence that will substantiate antibiotic stewardship, helping the optimization of preventative, diagnostic, and treatment protocols for bovine mastitis. Therefore, detailed information on the effect of treatment of *Escherichia coli* mastitis with third-generation cephalosporins is indispensable.

Our group has recently described the dynamics of milk microbiome upon antimicrobial treatment with ceftiofur in animals naturally infected with mastitis pathogens [34]. In that study, cows from a commercial dairy farm were enrolled upon diagnosis of clinical mastitis and randomly allocated to receive extended intramammary therapy with ceftiofur, or to receive no treatment. We observed that in cows with mastitis caused by *E. coli*, treatment with ceftiofur did not offer an advantage when compared to no treatment in terms of clinical cure, pathogen clearance rate, or bacterial load. Moreover, the milk microbiome from the affected quarters was indistinguishable from the adjacent healthy quarters within 14 days following the onset of the disease regardless of intramammary antimicrobial administration.

Herein, we describe a challenge model using a known strain of mastitis-causing *E. coli* to characterize the microbiome before, during, and after intramammary infection in a controlled setting. We aim to investigate the changes that occur upon introduction of a major pathogen and the ability of the healthy mammary microbiota to restore equilibrium with or without external aid of antimicrobials. Moreover, the role of antimicrobials in the normal milk microbiota has not yet been investigated in controlled longitudinal studies. We hypothesize that intramammary administration of a broad-spectrum antimicrobial might favor the overgrowth of specific organisms and incite a shift in the microbial profile of milk from normal quarters.

Therefore, the objectives of this study were to generate knowledge on the complex microbial ecology and treatment of mastitis, more specifically (a) describe the milk microbiome before, during, and after the infection of bovine mammary glands with a strain of *E. coli* previously isolated from a mastitis case; (b) compare microbial populations between infected and non-infected quarters; and (c) evaluate the impact of a third-generation cephalosporin on both healthy and mastitic milk.

## Methods

### Challenge strain

The strain used in this study (*E. coli* ECC-Z [35], Cornell University), hereafter referred as C1, was isolated from a clinical case of bovine mastitis, and was proven effective in previous experimental challenges to result in mild to moderate cases of clinical mastitis [36, 37]. Before an experimental challenge, frozen stocks of the strain were activated in Luria-Bertani (LB) broth, incubated at 37 °C for 12 h and subsequently streaked on a McConkey plates for *E. coli* colony isolation. DNA extraction was performed in isolated colonies using a QIAamp DNA minikit (Qiagen Inc., Valencia, CA), and a fragment of the 16S rRNA gene was amplified using the primers 27F (5′-AGAGTTTGATCMTGGCTCAG-3′) and 1492R (5′-ACCTTGTTACGACTT-3′), followed by sequencing at the Cornell University Core Laboratories Center (Ithaca, NY) through Sanger sequencing for confirmation of the identity of the isolate.

### Animal selection and housing

Twelve mature (second or greater lactation) Holstein cows were selected from the Cornell Veterinary Medicine Teaching Dairy (Ithaca, NY). Six animals, two at a time, were challenged during March of 2014, and six animals were challenged two at a time between March and April of 2015. Experimental challenge and sampling took place at the Large Animal Teaching and Research Unit (LARTU), Department of Animal Sciences, Cornell University (Ithaca, NY). Selected animals had no previous cases of clinical mastitis in the current lactation, were between 246 and 461 days in milk, and had an average somatic cell count of 95,000 cells/mL, ranging from 41,000 to 191,000 cells/mL, measured 1 week prior to transportation to LARTU.

Animals were housed individually in stalls bedded with sawdust and were fed ad libitum the same diet provided at the farm from which they were sourced, calculated to meet or exceed the requirements for lactating Holstein cows with a body weight of 650 kg producing 45 kg of 3.5% fat-corrected milk. The feed was transported daily from the Cornell Teaching Dairy. Animals were milked twice daily, at 08:00 and 20:00.

### Sampling procedures and experimental infection

**Fig. 1:**
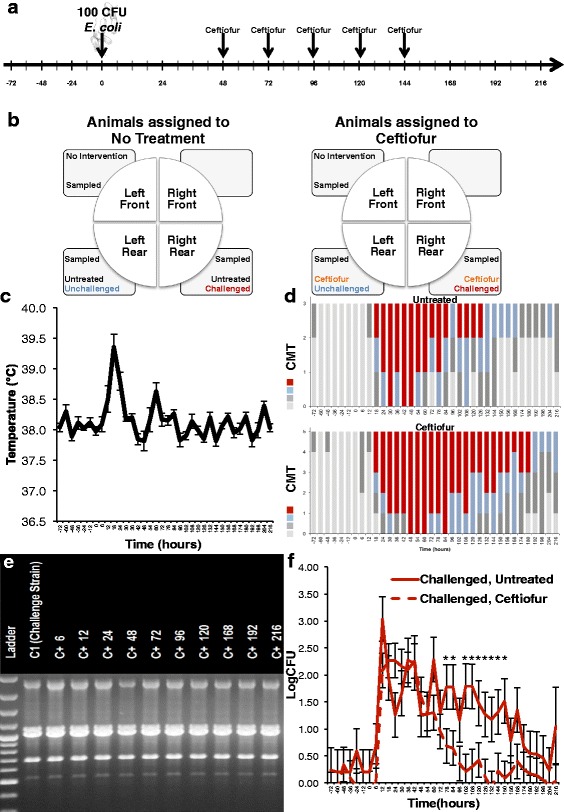
Experimental challenge timeline (**a**). Schematic of challenge and treatment in each quarter (**b**). Effect of intramammary infection with *E. coli* and treatment with ceftiofur hydrochloride (48, 72, 96, 120, and 144 h) on temperature (**c**). California Mastitis Test (CMT) results (**d**). RAPD strain typing results. (**e**) Effect of intramammary infection with *E. coli* and treatment with ceftiofur hydrochloride (48, 72, 96, 120, and 144 h) on colony-forming units (CFU) (**f**) Asterisks represent differences after Tukey-Kramer multiple comparison correction and α=0.05 between groups within the same time point

Sampling scheme, treatment allocation, and experimental design are depicted in (Fig. 1a, b). Milk samples were collected every 12 h during the 3 days that preceded intramammary infection with *E. coli* strain C1, henceforth indicated as time 0, and every 6 h from live challenge with *E. coli* until the ninth day after infection, with the last time point being at 216 h post-challenge. Only one quarter was challenged: 100 colony-forming units (CFU) of *E. coli* C1 suspended in 1 mL of phosphate buffered saline (PBS) solution was deposited immediately ventral of the Furstenberg’s rosette via the teat canal.

Each animal had three quarters sampled at each time point: the challenged quarter, an ipsilateral unchallenged quarter that was included in the same antibiotic treatment group as the challenged one and a third quarter which did not undergo any intervention (i.e., no challenge, no treatment) and was sampled in every time point as a within-animal control. Before milk sample collection, each cow had teats dipped in iodine (Bovadine sanitizing teat dip, DeLaval Manufacturing, Kansas City, MO), which was left in contact for at least 30 s. Teats were wiped with dry sterile gauze and final teat disinfection was performed with gauze soaked in 70% (*v*/*v*) ethanol immediately prior to collection of the milk sample. The three initial streams of milk were discarded; the milk was collected into sterile tubes in three different aliquots and immediately placed on ice. The first aliquot was collected and kept frozen at −20 °C until DNA extraction, the second aliquot was used for CFU counting, and the third aliquot was submitted for SCC determined through flow cytometry (Fossomatic FC, Eden Prairie, MN) at the Dairy One Cooperative Inc. (Ithaca, NY). Linear score (LS) was calculated based on SCC according to the equation LS= [ln(SCC/10^5^)/ln(2)] + 3. The health status and temperature of each cow were assessed at each sampling time, and cows showing signs of systemic illness were provided with appropriate supportive therapy, which included intravenous administration of fluids and intramuscular administration of non-steroidal anti-inflammatory drugs.

### Treatment administration

At 48 h after infection, animals were randomly allocated into either the treatment group, which received five consecutive intramammary infusions of ceftiofur hydrochloride comprised of 125 mg of ceftiofur equivalents (as ceftiofur hydrochloride; Spectramast LC®, Zoetis, Florham Park, NJ) at 24-h intervals in both challenged and ipsilateral unchallenged quarters, or the untreated control group, for which no substance was introduced into the mammary glands. The timing of the first treatment was chosen in an attempt to mimic the dynamics of culture-based treatment of coliform mastitis in a commercial dairy farm, and the first dose was administered immediately after sample collection of the 48-h sample.

### CFU counting and strain typing

Milk samples collected at time points following intramammary infection were inoculated on MacConkey agar in 100-μL aliquots and incubated at 37 °C overnight for bacterial identification. Total CFU per milliliter was calculated based on quantitative culture of serial milk dilutions in triplicates by averaging the number of colonies in the triplicate and multiplying the number by the dilution factor.

Strain typing was performed through random amplification of polymorphic DNA (RAPD) with primers designed specifically for RAPD typing of Gram-negative bacteria (forward 5′-AGTAAGTGACTGGGGTGAGCG-3′, reverse 5′TACATTCGAGGACCCCTAAGTG-3′), which have been shown to discriminate between different strains of mastitis-causing *E. coli* [38]. PCR products were evaluated for banding pattern using gel electrophoresis in a 1.5% agarose gel at 60 V for 1.5 h.

### DNA isolation and purification

Genomic DNA extraction was performed using the PowerFood DNA Isolation Kit (MO BIO Laboratories Inc., Carlsbad, CA), following the manufacturer’s recommendations with an extra incubation at 65 °C for 10 min prior to cell disruption to maximize DNA yields [39]. A 6-mL aliquot of milk was divided into fat, whey, and pellet through centrifugation. The whey was discarded, and the fat layer and pellet were used as starting sample in DNA extraction, as described previously [40]. Concentration and purity of isolated DNA were evaluated based on optical density at 230-, 260-, and 280-nm wavelengths (NanoDrop ND-1000, NanoDrop Technologies, Wilmington, DE).

### 16S rRNA gene amplification, MiSeq sequencing, and bioinformatic analyses

Isolated genomic DNA was used as a template for amplification of the V4 hypervariable region of the bacterial 16S rRNA gene using the primers 515F and 806R, which had been optimized for the Illumina MiSeq platform (Illumina Inc., San Diego, CA) [41] as described previously [42].

Six runs of the Illumina MiSeq sequencer were needed for sequencing of all samples. In each run, 279 samples and a sequencing control were pooled in an equimolar library and sequenced using the MiSeq reagent kit V2 for 300 cycles. Bioinformatics was performed using quality-filtered indexed reads, which were concatenated into a single FASTA file and uploaded in the open-source pipeline Quantitative Insights into Microbial Ecology (QIIME) version 1.9.1 [43], using computer resources of the Cornell Boyce Thompson Institute (Ithaca, NY). Sequences were handled following the default settings of the pipeline. Quality filtering was performed as described previously [44]. Open-reference taxonomic assignment into operational taxonomic units (OTUs) with 97% identity was achieved using UCLUST [45], RDP classifier [46], PyNAST [47], and the Greengenes [48] reference database. Rare OTUs with less than five sequences in each run, and samples with less than 1000 reads were excluded from the database. Within-sample diversity (α-diversity) was assessed through Shannon diversity index calculated in a randomly selected subset of the OTU database obtained through the script single_rarefaction.py in QIIME at a rarefication level of 1500 reads per sample. Between-sample microbial diversity (β-diversity) was assessed through phylogenetic-based weighted UniFrac [49] distances, calculated in QIIME through the script beta_diversity.py and the distance matrix obtained was used for comparison between groups.

### Statistical analyses

The UNIVARIATE procedure of SAS version 9.4 (SAS Institute Inc., Cary, NC) was used to perform descriptive analyses. Non-normally distributed variables (i.e., SCC and CFU) were normalized through log transformation. Longitudinal changes in the microbial profile was assessed through description of the relative abundances of the 25 most abundant bacterial families using the tabulate function of JMP Pro 12 (SAS Institute Inc., Cary, NC), and relative abundances of all the remaining families were combined into a single cluster, defined as “Other.” Variables of interest were evaluated between challenged, unchallenged, treated, and untreated quarters with repeated measures ANOVA using the GLIMMIX procedure of SAS. Significance of pairwise comparisons were adjusted through the Tukey-Kramer multiple comparison correction. Outcomes were the relative abundance of *Enterobacteriaceae*, Shannon diversity index, LogSCC, LS, and LogCFUs; explanatory variables were challenge (challenged versus control quarter), treatment (treated versus untreated quarter), time relative to experimental challenge, and their two- and three-way interactions.

To assess the effect of treatment, stratified analysis of covariance was performed in challenged and unchallenged quarters separately. To account for possible differences that occurred between intramammary infection and first treatment at 48 h, the average of values observed between challenge and treatment (i.e., 0, 6, 12, 24, 36, 42, and 48 hours relative to challenge) was included as a covariate in these models. Variables of interest were the relative abundance of *Enterobacteriaceae*, Shannon diversity index, LogSCC, LS, and LogCFUs; explanatory variables were treatment (treated versus untreated quarter), time relative to experimental challenge, and their two-way interactions. Teat nested within a cow was considered a random effect in all statistical models. The first-order ante-dependence covariance structure was selected because it resulted in the smallest Schwarz’s Bayesian information criterion value. Differences with *P* ≤ 0.05 were considered significant. Descriptive analyses of sequencing results are presented as average and standard deviation. All other results are presented as the least-square means followed by the respective standard error of the mean.

Three animals (animals E, G, and J—Additional file 1) experienced elevated SCC on 3 days preceding intramammary infusion of bacteria and for that reason did not develop an infection following the challenge with *E. coli*. A fourth animal (animal L—Additional file 1) acquired a natural intramammary infection in one of the unchallenged quarters caused by an *E. coli* strain different from the experimental challenge strain. Data collected from these four animals was only used to compare challenged, infected versus challenged, uninfected quarters. All other analyses assessing the effect of experimental infection and treatment with ceftiofur did not include data from animals E, G, J, and L.

Data were handled as follows: samples without a SCC value due to clotted milk (i.e., clinical mastitis) received a value of 30,000,000 SCC; samples with a CFU value that indicated too numerous to count received a value of 60,000 CFU. The rationale for choosing these arbitrary values was to assign a number that was larger than the largest observation for that variable in the dataset (i.e., the largest SCC observed was 27,255,000 and the largest CFU observed was 58,000).

Multivariate analysis of milk microbiome was implemented in QIIME and R (R Core Team, Vienna, Austria) [50]. Analysis of similarities (ANOSIM) was performed in non-rarefied data using the vegan [51] package in R. Groups shown to be significantly different in ANOSIM underwent Analysis of Composition of Microbiomes (ANCOM) [52] carried out in QIIME version 2.0.6 [53], in an attempt to identify which OTUs were driving the main differences between groups. Microbiome changes over time in challenged and control quarters were visualized through principal coordinates analysis (**PCoA**) of weighted UniFrac distances calculated in QIIME and plotted using EMPeror [54].

## Results

### Health characteristics

All cows exhibited elevated rectal temperature (mean 39.1 °C, StdDev 0.9 °C) at 18 h post-challenge (Fig. 1c); one animal had recurrent fever and received support therapy as described above.

Abnormal milk was observed within 18 h of infection (i.e., presence of flakes, clots, or serous milk) and lasted until 132 and 192 h in control and ceftiofur groups, respectively. Mild clinical signs of mammary inflammation were observed, which included abnormal milk and/or redness and swelling of the challenged quarters (Fig. 1d).

### Bacterial isolation and strain typing

The C1 strain of *E. coli* used for intramammary infection was isolated from milk samples collected post-challenge from every challenged quarter, except the three quarters that presented elevated SCC on the days preceding intramammary infusion of bacteria, which were excluded from further analysis. Confirmation of strain was performed through RAPD strain typing (Fig. 1e).

### Effect of intramammary infection with *E. coli* and intramammary antimicrobial therapy on CFU results

Intramammary infection with *E. coli* significantly increased LogCFUs (*P* < 0.0001); intramammary treatment with ceftiofur significantly decreased LogCFUs in challenged, infected quarters (*P* < 0.0001). Tukey-adjusted comparisons in each sampling time revealed that ceftiofur-treated quarters had significantly lower LogCFUs at 78, 84, 102, 108, 120, 126, 132, 144, and 150 h relative to challenge (Fig. 1f). Nevertheless, bacterial growth in samples collected from both control and treated quarters presented a decrease in CFU counts over time.

### Effect of intramammary infection with *E. coli* on somatic cell count measured as linear scores

**Fig. 2:**
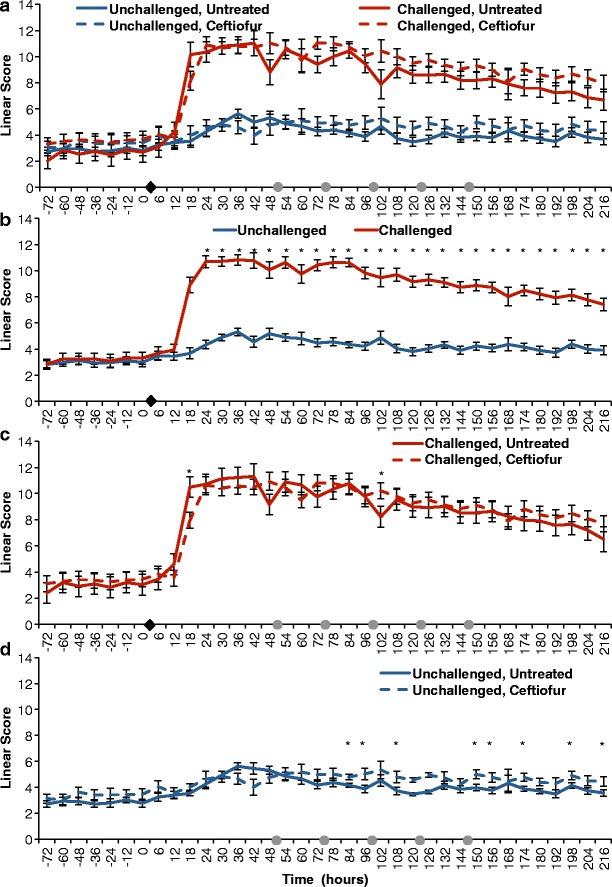
Effect of experimental infection with *Escherichia coli* (0 h) and intramammary treatment with ceftiofur hydrochloride (48, 72, 96, 120, and 144 h) on Linear Scores. Effect of challenge and treatment (**a**, *N* = 24 quarters), effect of intramammary challenge with *Escherichia coli* (**b**, *N* = 24), stratified analysis of covariance assessing the effect of intramammary treatment with ceftiofur hydrochloride in challenged (**c**, *N* = 8), and unchallenged quarters (**d**, *N* = 16). *Asterisks* represent differences after Tukey-Kramer multiple comparison correction and *α* = 0.05 between groups within the same time point. *Black diamonds* represent experimental infection with 100 CFU of *E. coli*, and *grey circles* represent intramammary treatment with ceftiofur

Intramammary infusion with 100 CFU of *E. coli* increased SCC as early as 6 h post-challenge, peaking around 18 h and remaining significantly higher when compared to unchallenged quarters until the end of the study period (*P* < 0.0001) (Fig. 2b). A non-significant increase in somatic cell count on unchallenged quarters was also observed between 12 and 48 h relative to challenge (Fig. 2d). Finally, intramammary treatment with ceftiofur hydrochloride did not significantly decrease linear score throughout treatment, with only two time points exhibiting different linear scores between treated and untreated groups (Fig. 2c).

### Sequencing results

A total of 53,019,538 sequences passed quality control and were available for downstream analysis. The dataset analyzed, which only included infected animals, comprised 34,193,997 reads with a mean of 39,622 and standard deviation of 31,034 reads per sample.

### Taxonomic classification

On average, only 22.2% of all reads were not classified at the family level (StdDev 19.3%), whereas the number of unclassified reads at the genus level was on average 50.6% (StdDev 23.7%). The most abundant families in the dataset were *Ruminococcaceae* (mean 13.5%, StdDev 12.0%) *Enterobacteriaceae* (mean 13.4%, StdDev 24.6%), *Aerococcaceae* (mean 5.6%, StdDev 7.7%), *Lachnospiraceae* (mean 5.4%, StdDev 5.4%), *Corynebacteriaceae* (mean 5.2%, StdDev 6.5%), *Planococcaceae* (mean 5.2%, StdDev 7.9%), *Bacillaceae* (mean 4.7%, StdDev 5.9%), *Clostridiaceae* (mean 4.5%, StdDev 3.9%), *Bacteroidaceae* (mean 4.2%, StdDev 3.6%), and *Staphylococcaceae* (mean 3.6%, StdDev 6.5%). Detailed information on bacterial profile per study animal is provided in Additional file 1.

### Pre-challenge microbial profile

**Fig. 3:**
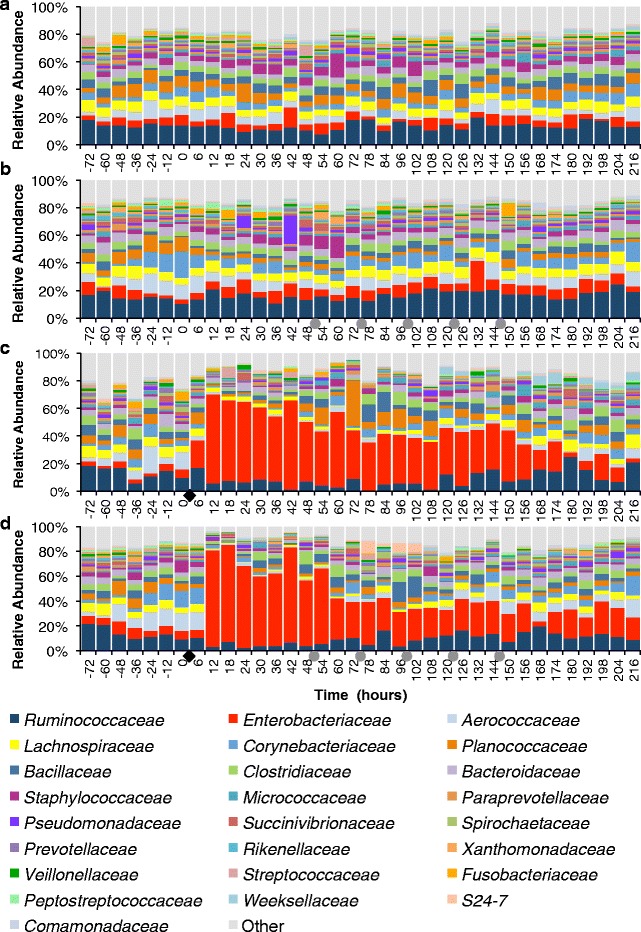
Effect of intramammary infection with *E. coli* and subsequent treatment with ceftiofur hydrochloride (48, 72, 96, 120, and 144 h) on relative abundance of the 25 most prevalent families in unchallenged untreated quarters (**a**, *N* = 11), in unchallenged ceftiofur-infused quarters (**b**, *N* = 5), in challenged untreated quarters (**c**, *N* = 3), and in challenged and ceftiofur-treated quarters (**d**, *N* = 5). *Black diamonds* represent experimental infection with 100 CFU of *E. coli*, and *grey circles* represent intramammary treatment with ceftiofur

The microbial profile prior to intramammary infusion of *E. coli* (−72 to 0 h) was diverse (Fig. 3). No differences were observed between challenged, unchallenged, treated, and untreated groups in the pre-challenge microbiome. The most abundant families in pre-challenge samples were *Ruminococcaceae* (mean 16.8%, StdDev 10.1%), *Lachnospiraceae* (mean 7.0%, StdDev 5.1%), *Aerococcaceae* (mean 6.8%, StdDev 8.2%), *Enterobacteriaceae* (mean 6.3%, StdDev 13.5%), *Planococcaceae* (mean 5.7%, StdDev 9.5%), *Bacteroidaceae* (mean 5.4%, StdDev 3.3%), *Corynebacteriaceae* (mean 5.1%, StdDev 7.3%), *Clostridiaceae* (mean 4.2%, StdDev 3.1%), *Bacillaceae* (mean 3.5%, StdDev 3.7%), and *Staphylococcaceae* (mean 2.8%, StdDev 4.9%).

### Effect of experimental infection with *E. coli* and intramammary antimicrobial therapy on milk microbiome

**Fig. 4:**
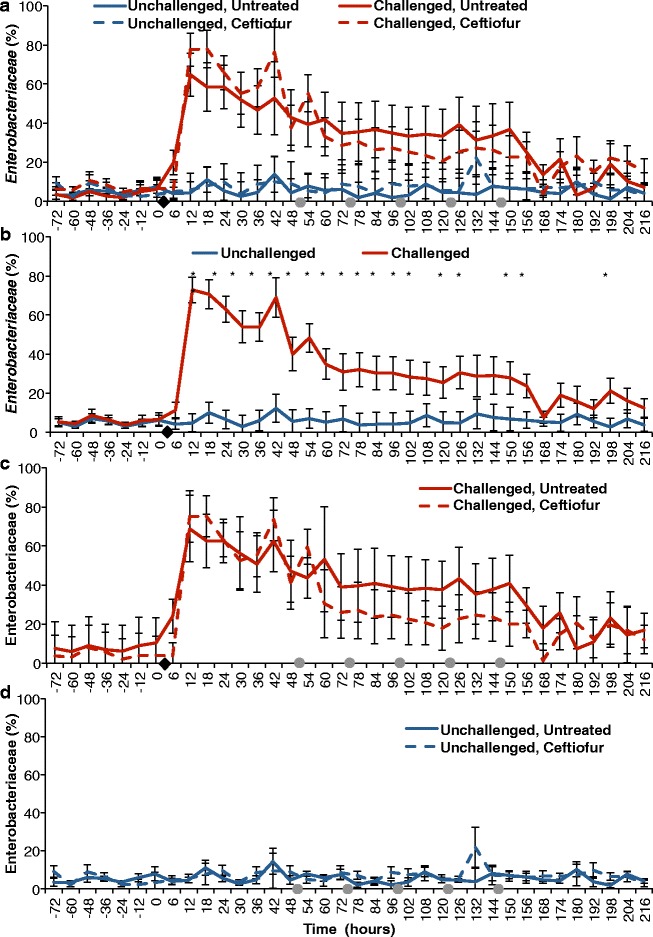
Effect of experimental infection with *Escherichia coli* (0 h) and intramammary treatment with ceftiofur hydrochloride (48, 72, 96, 120, and 144 h) on relative abundance of *Enterobacteriaceae.* Effect of challenge and treatment (**a**, *N* = 24 quarters), effect of intramammary challenge with *Escherichia coli* (**b**, *N* = 24) and stratified analysis of covariance assessing the effect of intramammary treatment with ceftiofur hydrochloride in challenged (**c**, *N* = 8) and unchallenged quarters (**d**, *N* = 16). *Asterisks* represent differences after Tukey-Kramer multiple comparison correction and *α* = 0.05 between groups within the same time point. *Black diamonds* represent experimental infection with 100 CFU of *E. coli*, and *grey circles* represent intramammary treatment with ceftiofur

Intramammary infection with *E. coli* dramatically changed the milk microbial profile. Before challenge, quarters presented a very diverse profile, with the family *Ruminococcaceae* being the most abundant, averaging 13.3 and 14.3% in challenged untreated and challenged ceftiofur groups, respectively (Fig. 3c, d). After experimental infection with *E. coli*, the milk microbiome had a significant increase in the family *Enterobacteriaceae* (*P* < 0.0001) (Fig. 4b), which represented over 30% of the relative abundance from 12 to 150 h, peaking at 64.7% at 12 h post-challenge in the untreated group (Fig. 3c). Likewise, animals that eventually received intramammary treatment had *Enterobacteriaceae* as the predominant group, representing over 30% of the relative abundance from 12 to 60 h, peaking at 77.9% at 18 h (Fig. 3d). Intramammary treatment with ceftiofur hydrochloride did not significantly improve the clearance rate of *Enterobacteriaceae*, nor significantly decreased the relative abundance of *Enterobacteriaceae* in any time point when compared to challenged untreated quarters (Fig. 4c).

The microbial profile of unchallenged quarters was more diverse than what was observed in the post-infection challenged quarters (Fig. 3). There was no remarkable change in the relative abundances of the 25 most abundant families with intramammary treatment of unchallenged quarters with ceftiofur (Fig. 3b).

### Effect of experimental infection with *E. coli* and intramammary antimicrobial therapy on bacterial diversity

**Fig. 5:**
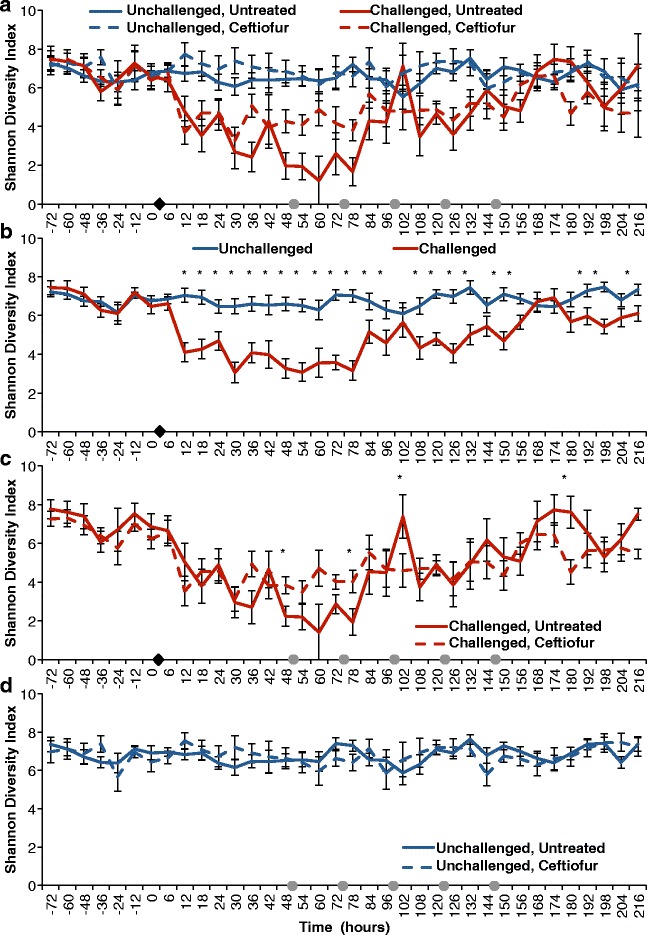
Effect of experimental infection with *Escherichia coli* (0 h) and intramammary treatment with ceftiofur hydrochloride (48, 72, 96, 120, and 144 h) on Shannon diversity index. Effect of challenge and treatment (**a**, *N* = 24 quarters), effect of intramammary challenge with *Escherichia coli* (**b**, *N* = 24) and stratified analysis assessing the effect of intramammary treatment with ceftiofur hydrochloride in challenged (**c**, *N* = 8) and unchallenged quarters (**d**, *N* = 16). Asterisks represent differences after Tukey-Kramer multiple comparison correction and α = 0.05 between groups within the same time point. *Black diamonds* represent experimental infection with 100 CFU of *E. coli*, and *grey circles* represent intramammary treatment with ceftiofur

Shannon diversity index was high and similar in all groups previous to experimental infection (Fig. 5a). Diversity levels sharply decreased after experimental challenge with *E. coli* (*P* < 0.0001), and the lowest diversity was observed between 30 and 78 h post-infection (Fig. 5a, b). Administration of five doses of ceftiofur in 24-h intervals starting at 48 h post-infection did not alter bacterial diversity in a consistent manner; differences were observed in the diversity indexes before initiation of treatment regimen (at 36 and 48 h). Nevertheless, Tukey-adjusted comparisons revealed that ceftiofur-treated animals had significantly different diversity indexes at 78, 102, and 180 h relative to challenge (Fig. 5c). By the end of the study period, treated and untreated quarters did not present significantly different diversity (Fig. 5c).

Unchallenged quarters presented high and stable diversity indexes throughout the study (Fig. 5a, d). No difference was observed in diversity levels in unchallenged quarters between control and ceftiofur-infused quarters.

### Effect of pre-challenge linear scores on intramammary infection with *E. coli*

**Fig. 6:**
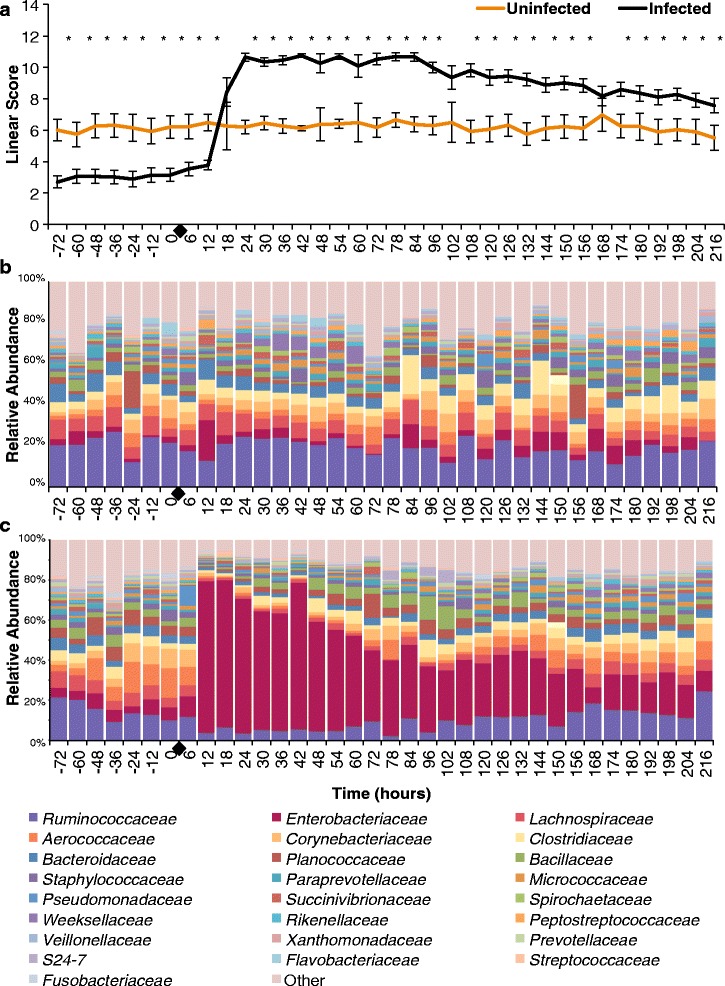
Effect of pre-challenge linear scores on intramammary infection success (**a**). Depiction of the relative abundance of the 25 most prevalent families in challenged uninfected quarters (**b**, *N* = 3), and challenged and infected quarters (**c**, *N* = 8). *Black diamonds* represent experimental infection with 100 CFU of *E. coli*

Animals that were challenged with 100 CFU of *E. coli* and were successfully infected had significantly lower linear scores in all time points preceding challenge when compared to cows that were found not to be infected with the strain C1 (Fig. 6a). Animals infected with the challenge strain presented a sharp rise in LS as soon as 18 h after challenge and sustained higher linear scores throughout the study period when compared to challenged and uninfected quarters (Fig. 6a). The microbial profile of uninfected quarters only had an increase in the relative abundance of the family *Enterobacteriaceae* representing 19% of the microbial profile at the 12-h time point post-challenge (Fig. 6b), whereas infected quarters had their milk microbiome dominated by the family *Enterobacteriaceae* which represented over 75% of the relative abundance at the 12-h time point post-challenge and accounted for over 30% of the microbial profile until 132-h post-challenge (Fig. 6c).

### Multivariate analysis of milk microbiome and effect of intramammary antimicrobial therapy

**Fig. 7:**
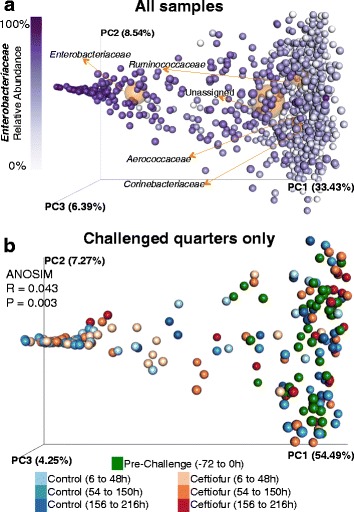
Biplot depicting weighted UniFrac distances of all samples and the coordinates of the five most abundant family-level taxa (*orange spheres*) in the context of relative abundance of *Enterobacteriaceae* (**a**). Analysis of similarity (ANOSIM) and principal coordinate analysis (PCoA) of weighted UniFrac distances comparing the effect of challenge, treatment and time in challenged quarters only (**b**). For comparison and plotting purposes, time points were discretized into seven categories

The relative abundance of the family *Enterobacteriaceae* was the main driver of the variation on weighted UniFrac distances in the dataset comprised of all samples from infected cows (Fig. 7a). Samples from challenged quarters were discretized into seven categories according to time relative to the experimental challenge, as well as the treatment group. A significant difference in the milk microbiome was detected between the seven categories in ANOSIM, which was corroborated by a clustering observed in weighted UniFrac PCoA (Fig. 7b). Analysis of composition of microbiomes identified ten OTUs that significantly differed between groups, four of which were assigned to the family *Enterobacteriaceae*, one was assigned to the family *Planococcaceae*, and five OTUS were not taxonomically assigned at the family level (Additional file 2).

**Fig. 8:**
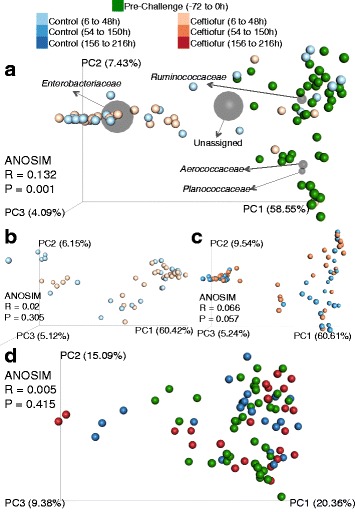
Multivariate analysis of milk microbiome. Effect of intramammary infection with *E.* coli on UniFrac distances and depiction of the five most abundant family-level taxa. Analysis of similarity (ANOSIM) and principal coordinate analysis (PCoA) of weighted UniFrac distances comparing pre-challenge (−72 to 0 h) and post-challenge (6 to 48 h) samples (**a**). Weighted UniFrac distances and ANOSIM comparing control and ceftiofur before initiation of treatment (**b**) and during treatment (**c**). ANOSIM comparing pre-challenge (−72 to 0 h) samples to control and ceftiofur samples after cessation of treatment (156 to 216 h) (**d**). For comparison and plotting purposes, time points were discretized into categories

Stratified ANOSIM revealed a significant difference when the microbiome pre-challenge (−72 to 0 h) was compared to post-challenge pre-treatment (6 to 48 h). Grouping was observed in weighted UniFrac PCoA, with the family *Enterobacteriaceae* being the main driver of variation in this comparison (Fig. 8a). Seven OTUs were deemed significantly different between groups in ANCOM, two of which were assigned to the family *Enterobacteriaceae*, and five of which were not taxonomically assigned at the family level (Additional file 3).

The microbiome of samples from challenged treated and challenged untreated quarters were compared through ANOSIM before initiation of treatment (6 to 48 h) and during treatment administration (54 to 150 h). No differences were detected between treatment and control group in either ANOSIM or weighted UniFrac PCoA before initiation of treatment (Fig. 8b) or during treatment administration (Fig. 8c). When the microbiome of pre-challenge samples was compared to the of samples collected after treatment cessation (156 to 216 h) through ANOSIM no significant differences were identified, which was corroborated through the lack of grouping in weighted UniFrac PCoA (Fig. 8d). In an attempt to identify if quarters that were not successfully infected after experimental infection had a different pre-challenge microbiome from quarters that were successfully infected, we performed ANOSIM in pre-challenge samples. No difference was observed on the milk microbiome prior to experimental infection between challenged infected and challenged uninfected quarters (ANOSIM R = −0.17, *P* = 0.99). In agreement with ANOSIM, PCoA of weighted UniFrac distances did not reveal any clustering pattern (data not shown). All samples from an individual animal were sequenced in order within a run; batch effects that could have arisen due to samples being sequenced in six different runs were examined through weighted UniFrac PCoA, and no clustering due to sequencing run was observed (data not shown).

## Discussion

We have used an in vivo experimental model of bovine mastitis and state-of-the-art technology to describe in detail the dynamic changes that the milk microbiome undergoes upon infection, treatment, and resolution of mastitis. To the best of our knowledge, this is the first study to investigate an experimental challenge of bovine mastitis using next-generation sequencing, and the first investigation on the effects of third-generation cephalosporins on the endogenous microbiota of healthy milk. Here, we show that extended intramammary treatment with ceftiofur has no effect on the microbiome of milk from *E. coli*-induced mastitis. Using multivariate analysis of weighted UniFrac distances and ANOSIM, we demonstrate that the milk microbiome returns to a similar state to that of unchallenged quarters 9 days after experimental intramammary infection with *E. coli*, regardless of receiving antimicrobial therapy. Our results show a significant decrease on the LogCFUs recovered from milk samples in challenged and treated quarters; however, no beneficial effect of antimicrobial treatment was observed in somatic cell count, rate of decrease of *Enterobacteriaceae*, or microbial diversity in quarters challenged with *E. coli*.

We observed a dramatic decrease in microbial diversity following the experimental challenge with 100 CFU of *E. coli*. Other studies that investigated infections in both human [55] and bovine milk [28, 29, 33] have also reported that reduced microbial diversity was associated with mastitis. Interestingly, we observe here that diversity indexes of challenged quarters returned to indexes comparable to uninfected quarters by the end of the study period. This is similar to the results observed by our group in a study that investigated the microbiome of animals naturally infected with mastitis [34]. It is important to highlight that experimental infections such as the one carried out in this study are performed in a limited number of animals because of ethical and monetary reasons; therefore, it is possible that statistically significant differences could not be identified here because of the lack of power.

Treatment of challenged quarters with ceftiofur significantly decreased LogCFUs; however, this effect could only be observed during treatment administration, with no significant differences in LogCFUs detected 12 h after the last intramammary infusion (156-h post-challenge). These findings are in agreement with our previous study [34] that investigated the effect of ceftiofur in animals naturally infected with mastitis. In that study, we were able to identify a significant reduction in bacterial load of treated animals as measured through a number of 16S rRNA copies during treatment administration; on the other hand, we failed to identify any differences in bacterial load after cessation of treatment. Despite the significant drop in LogCFUs observed in treated challenged quarters, we could not identify a treatment effect in the relative abundance of *Enterobacteriaceae.* Taking in consideration the significant reduction in live bacteria detected in the present study, it is possible that the relative abundance of bacteria remains unchanged but treatment with ceftiofur has an effect in impairing the viability of bacteria exposed to the antimicrobials.

Herein, we were unable to recognize a treatment effect neither on the rate of change of *Enterobacteriaceae* nor in the overall microbial profile of challenged quarters. These findings are in agreement with our previous work [34], in which no significant differences were observed in the rate of decrease of *Enterobacteriaceae* in animals naturally infected with *E. coli*. Moreover, we failed to identify a significant effect of treatment on the total milk microbiome as measured by ANOSIM, which is in line with the results from our earlier investigation. Several studies have investigated the effect of ceftiofur in food animals [56–58]; however, these studies evaluated parenteral or oral administration of ceftiofur in different microbiome niches, such as the gut. To the best of our knowledge, our group is the first to evaluate the effect of intramammary ceftiofur in bovine milk. The effect of chemotherapy in human milk microbiome and metabolome has been previously reported [59]. In bovine milk, modifications in the metabolome have also been described after the use of parenteral enrofloxacin [60]. The evidence of ceftiofur-induced modifications in the microbiome of food animals and the indication of possible drug-induced changes in milk prompt further investigation to simultaneously characterize the effects of cephalosporins in the mammary gland microbiome, metabolome, and resistome. It is possible that the observed lack of change in the relative abundance could be accompanied by differences in the metabolic profile of bacteria exposed to antimicrobials. Using technologies such as shotgun metagenomics, metabolomics, and proteomics [61], we could detect differences not evident by 16S rRNA sequencing, and conclusions about the effects of cephalosporins in the diseased mammary gland may change.

In our study we observed a dramatic change on the milk microbial profile upon infection with *E. coli*. Nevertheless, the most remarkable result of this investigation was the lack of discrimination between the microbiome of pre-challenge samples and the microbiome of milk samples from the same quarters collected after disappearance of clinical signs. Multivariate analysis of milk microbiome identified a significant difference when pre-challenged samples were compared to the ones collected in the first 2 days after challenge (−72 to 0 h versus 6 to 48 h). However, we failed to identify any differences between the microbiome of treated and untreated quarters during treatment administration (time points 54 to 150). Interestingly, no difference could be observed in the overall microbiome assessed through ANOSIM between groups after treatment cessation, indicating that the milk microbiome is capable of returning to the original microbial status. The restoration of the microbiota to a healthy milk profile is in line with the findings of our investigation in naturally infected animals, in which no differences on the microbiome of healthy quarters and cured quarters could be observed 14 days after diagnosis of mastitis. Conversely, work by Falentin et al., [32] identified long-lasting effects in quarters with normal milk that had a history of mastitis and suggested that such effects could be due to clinical mastitis and the antimicrobials used for the treatment thereof. Nevertheless, work performed by that group consisted of a cross-sectional study, and several components related to study design, sample collection, and the pathogens associated with mastitis in those animals could contribute for differences in the findings between the two studies. Those authors identified high abundance of members of the family *Staphylococcaceae*, with some samples having as much as 30% of staphylococci reads assigned to *S. aureus*, which is known to have adapted to persist in the mammary environment, and attach to the cell lining [62]. Regardless of *S. aureus* and *E. coli* both being considered major mastitis pathogens, the mammary environment is known to be a reservoir of *Staphylococcus* while *E. coli* is mainly considered an environmental mastitis-causing bacteria [62].

Antimicrobial infusion in healthy mammary glands did not have a significant effect in the microbial profile. This was an interesting finding, given that alterations in the flora of healthy individuals exposed to antimicrobials favoring the blooming of pathogenic bacteria have been described [63]. Previous reports of mastitis outbreaks following “blitz” therapy [64], which consists in intramammary treatment of all lactating animals in the herd for elimination of a contagious pathogen, led us to hypothesize that shifts in the microbiome of healthy milk would occur in consequence to antimicrobial exposure, as it has been observed in other niches [58, 63]. Nevertheless, intriguing work by Zaura and colleagues [65] have reported a surprising resilience of the salivary microbiome upon exposure to different antimicrobials. While authors of the latter study observed significant and long-lasting changes in the fecal microbiome, the microbiome of the saliva presented only short-term ecological consequences, representing two radically different responses in two niches of the same individuals. It is possible that the microbiome of the mammary gland present itself as stable and resilient, comparable to what was observed in the salivary microbiome. This hypothesis is corroborated by the highly diverse microbiome of healthy milk described in many studies [61, 66, 67]. One could also speculate that the healthy milk microbiome does not contain strains of bacteria that are resistant to ceftiofur and capable of taking advantage due to the lack of competition imposed by antimicrobial exposure. On the contrary of what is observed in the gut, the very low bacterial load in healthy milk reported in our former study might indicate that the milk microbial environment is not as competitive and is less favorable to the overgrowth of bacteria and subsequent change in the microbial profile.

In this study, three out of the 12 challenged cows did not develop an infection following the challenge with *E. coli*. Several factors could account for this occurrence, most importantly the fact that these animals had significantly higher SCC in the time points prior to experimental infection. This finding is in agreement with Schukken [62] and Burvenich [68] which have reported that the success of any intramammary infection is dependent upon the stage of lactation and the initial amount of milk leukocytes. In addition, our data follows the reasoning of Burvenich [68], which stated that the severity and outcome of *E. coli* mastitis are cow-dependent, rather than entirely pathogen-dependent; however, it is important to highlight that in our study, we have evaluated infection with a single-characterized strain of *E. coli*. The dynamics of *E. coli*-associated mastitis and its resolution are multifactorial, involving aspects of the animal’s immune system and features of the pathogen involved [69, 70]. Due to the anatomic structure of the udder, it is generally assumed that infection in one quarter should not affect the immune status of neighboring quarters. Recent studies have contested this hypothesis, providing evidence of interdependence between infected and healthy quarters [71, 72]. Jensen and colleagues [73] have evaluated the transcriptional response of uninfected quarters in animals challenged with two major mastitis pathogens and described that the response in non-affected quarters was greater in animals with *E. coli*-associated mastitis [73]. Although other immune parameters were not evaluated in the current study, the numerical increase in somatic cell count in unchallenged quarters after intramammary infection with *E. coli* observed here is in agreement with Jensen et al., [73] and Blagitz et al., [71], indicating that the immune response in the mammary gland is to some extent influenced by the status of adjacent quarters.

## Conclusion

We have demonstrated here that the bovine mammary gland harbors a resilient microbiome, capable of reestablishing itself after dramatic changes due to an infectious event with an environmental pathogen. While all cows were inoculated with the same bacterial load, unique responses were observed in different animals. No differences were observed in the microbial profile of unchallenged mammary glands that were exposed to extended intramammary antimicrobial therapy. The milk microbiome was shown to be diverse and stable, indicating that bacteria within the mammary gland are tightly regulated. Our results corroborate for judicious use of antimicrobials in the dairy industry, demonstrating that due to the resilience of the mammary gland microbiome, certain cases of mastitis are capable of resolving independently the use of intramammary antimicrobials.

## Additional files

Additional file 1: Detailed information on bacterial profile per study. animal (PDF 888 kb)

Additional file 2: ANCOM results 7b. (XLSX 1094 kb)

Additional file 3: ANCOM results 8b. (XLSX 172 kb)

## References

[1] Heikkila AM, Nousiainen JI, Pyorala S (2012). Costs of clinical mastitis with special reference to premature culling. J Dairy Sci.

[2] Rollin E, Dhuyvetter KC, Overton MW (2015). The cost of clinical mastitis in the first 30 days of lactation: an economic modeling tool. Prev Vet Med.

[3] USDA (2016). Dairy 2014, Milk Quality, Milking Procedures, and Mastitis in the United States, 2014.

[4] Fogsgaard KK, Bennedsgaard TW, Herskin MS (2015). Behavioral changes in freestall-housed dairy cows with naturally occurring clinical mastitis. J Dairy Sci.

[5] Medrano-Galarza C (2012). Behavioral changes in dairy cows with mastitis. J Dairy Sci.

[6] Peters MD, Silveira ID, Fischer V (2015). Impact of subclinical and clinical mastitis on sensitivity to pain of dairy cows. Animal.

[7] Grohn YT (2004). Effect of pathogen-specific clinical mastitis on milk yield in dairy cows. J Dairy Sci.

[8] Bar D (2008). Effects of repeated episodes of generic clinical mastitis on mortality and culling in dairy cows. J Dairy Sci.

[9] Schukken YH (2009). Effects of repeated gram-positive and gram-negative clinical mastitis episodes on milk yield loss in Holstein dairy cows. J Dairy Sci.

[10] Pol M, Ruegg PL (2007). Treatment practices and quantification of antimicrobial drug usage in conventional and organic dairy farms in Wisconsin. J Dairy Sci.

[11] Jones GMS, J M. Environmental Streptococcal and Coliform Mastitis. Virginia Cooperative Extension; 2012. publication 404-234. https://www.pubs.ext.vt.edu/content/dam/pubs_ext_vt_edu/404/404-234/404-234_pdf.pdf.

[12] Bushnell RB (1984). The importance of hygienic procedures in controlling mastitis. Vet Clin North Am Large Anim Pract.

[13] Pal C (2016). The structure and diversity of human, animal and environmental resistomes. Microbiome.

[14] Ma L (2016). Metagenomic assembly reveals hosts of antibiotic resistance genes and the shared resistome in pig, chicken, and human feces. Environ Sci Technol.

[15] Pitta DW (2016). Metagenomic evidence of the prevalence and distribution patterns of antimicrobial resistance genes in dairy agroecosystems. Foodborne Pathog Dis.

[16] Muziasari WI (2016). The resistome of farmed fish feces contributes to the enrichment of antibiotic resistance genes in sediments below Baltic Sea fish farms. Front Microbiol.

[17] Lhermie G, Grohn YT, Raboisson D (2016). Addressing antimicrobial resistance: an overview of priority actions to prevent suboptimal antimicrobial use in food-animal production. Front Microbiol.

[18] Singer RS (2003). Antibiotic resistance—the interplay between antibiotic use in animals and human beings. Lancet Infect Dis.

[19] FDA, FDA’s CVM Key Initiatives for Antimicrobial Stewardship. 2017. https://www.fda.gov/AnimalVeterinary/SafetyHealth/AntimicrobialResistance/JudiciousUseofAntimicrobials/ucm535158.htm.

[20] Dar OA (2016). Exploring the evidence base for national and regional policy interventions to combat resistance. Lancet.

[21] The One Health Initiative. http://www.onehealthinitiative.com/.

[22] van Helden PD, van Helden LS, Hoal EG (2013). One world, one health. Humans, animals and the environment are inextricably linked—a fact that needs to be remembered and exploited in our modern approach to health. EMBO Rep.

[23] WHO. Critically important antimicrobials for human medicine. World Health Organization; 2012. 3rd rev. http://apps.who.int/iris/bitstream/10665/77376/1/9789241504485_eng.pdf.

[24] Schukken YH (2011). Randomized clinical trial to evaluate the efficacy of a 5-day ceftiofur hydrochloride intramammary treatment on nonsevere gram-negative clinical mastitis. J Dairy Sci.

[25] Suojala L, Kaartinen L, Pyorala S (2013). Treatment for bovine Escherichia coli mastitis—an evidence-based approach. J Vet Pharmacol Ther.

[26] Boix-Amoros A, Collado MC, Mira A (2016). Relationship between milk microbiota, bacterial load, macronutrients, and human cells during lactation. Front Microbiol.

[27] Hunt KM (2011). Characterization of the diversity and temporal stability of bacterial communities in human milk. PLoS One.

[28] Oikonomou G (2012). Microbial diversity of bovine mastitic milk as described by pyrosequencing of metagenomic 16s rDNA. PLoS One.

[29] Bhatt VD (2012). Milk microbiome signatures of subclinical mastitis-affected cattle analysed by shotgun sequencing. J Appl Microbiol.

[30] Lima SF (2017). The bovine colostrum microbiome and its association with clinical mastitis. J Dairy Sci.

[31] Quigley L (2013). The complex microbiota of raw milk. FEMS Microbiol Rev.

[32] Falentin H (2016). Bovine teat microbiome analysis revealed reduced alpha diversity and significant changes in taxonomic profiles in quarters with a history of mastitis. Front Microbiol.

[33] Oikonomou G (2014). Microbiota of cow’s milk; distinguishing healthy, sub-clinically and clinically diseased quarters. PLoS One.

[34] Ganda EK (2016). Longitudinal metagenomic profiling of bovine milk to assess the impact of intramammary treatment using a third-generation cephalosporin. Sci Rep.

[35] Dogan B (2012). Phylogroup and lpfA influence epithelial invasion by mastitis associated Escherichia coli. Vet Microbiol.

[36] Quesnell RR (2012). Bovine intramammary Escherichia coli challenge infections in late gestation demonstrate a dominant antiinflammatory immunological response. J Dairy Sci.

[37] Sipka A (2013). Prednisolone and cefapirin act synergistically in resolving experimental Escherichia coli mastitis. J Dairy Sci.

[38] Dogan B (2006). Adherent and invasive Escherichia coli are associated with persistent bovine mastitis. Vet Microbiol.

[39] Quigley L (2012). A comparison of methods used to extract bacterial DNA from raw milk and raw milk cheese. J Appl Microbiol.

[40] Ganda EK (2016). Evaluation of an on-farm culture system (Accumast) for fast identification of milk pathogens associated with clinical mastitis in dairy cows. PLoS One.

[41] Caporaso JG (2012). Ultra-high-throughput microbial community analysis on the Illumina HiSeq and MiSeq platforms. Isme J.

[42] Foditsch C (2015). Oral administration of Faecalibacterium prausnitzii decreased the incidence of severe diarrhea and related mortality rate and increased weight gain in preweaned dairy heifers. PLoS One.

[43] Caporaso JG, et al. QIIME allows analysis of high-throughput community sequencing data. Nat Methods. 2010;7(5):335–6. doi:10.1038/nmeth.f.303. 10.1038/nmeth.f.303 PMC3156573 20383131

[44] Bokulich NA (2013). Quality-filtering vastly improves diversity estimates from Illumina amplicon sequencing. Nat Methods.

[45] Edgar RC (2010). Search and clustering orders of magnitude faster than BLAST. Bioinformatics.

[46] McDonald D (2012). An improved Greengenes taxonomy with explicit ranks for ecological and evolutionary analyses of bacteria and archaea. ISME J.

[47] Caporaso JG (2010). PyNAST: a flexible tool for aligning sequences to a template alignment. Bioinformatics.

[48] DeSantis TZ (2006). Greengenes, a chimera-checked 16S rRNA gene database and workbench compatible with ARB. Appl Environ Microbiol.

[49] Lozupone C, Knight R (2005). UniFrac: a new phylogenetic method for comparing microbial communities. Appl Environ Microbiol.

[50] Team, R.C (2016). R: A language and environment for statistical computing.

[51] Oksanen J, Guillaume Blanchet F, Friendly M, Kindt R, Legendre P, McGlinn D, Minchin PR, O'Hara RB, Simpson GL, Solymos P, Stevens MHH, Szoecs E, Wagner H. vegan: Community Ecology Package. R package version 2.4-1. 2016. https://CRAN.R-project.org/package=vegan.

[52] Mandal S (2015). Analysis of composition of microbiomes: a novel method for studying microbial composition. Microb Ecol Health Dis.

[53] Caporaso JG (2010). QIIME allows analysis of high-throughput community sequencing data. Nat Methods.

[54] Vazquez-Baeza Y (2013). EMPeror: a tool for visualizing high-throughput microbial community data. Gigascience.

[55] Fernandez L (2013). The human milk microbiota: origin and potential roles in health and disease. Pharmacol Res.

[56] Fleury MA (2015). Impact of ceftiofur injection on gut microbiota and Escherichia coli resistance in pigs. Antimicrob Agents Chemother.

[57] Baron S (2016). Impact of the administration of a third-generation cephalosporin (3GC) to one-day-old chicks on the persistence of 3GC-resistant Escherichia coli in intestinal flora: an in vivo experiment. Vet Microbiol.

[58] Chambers L (2015). Metagenomic analysis of antibiotic resistance genes in dairy cow feces following therapeutic administration of third generation cephalosporin. PLoS One.

[59] Urbaniak C (2014). Effect of chemotherapy on the microbiota and metabolome of human milk, a case report. Microbiome.

[60] Junza A (2016). Metabolic profile modifications in milk after enrofloxacin administration studied by liquid chromatography coupled with high resolution mass spectrometry. J Chromatogr A.

[61] Addis MF (2016). The bovine milk microbiota: insights and perspectives from -omics studies. Mol Biosyst.

[62] Schukken YH (2011). Host-response patterns of intramammary infections in dairy cows. Vet Immunol Immunopathol.

[63] Theriot CM (2014). Antibiotic-induced shifts in the mouse gut microbiome and metabolome increase susceptibility to Clostridium difficile infection. Nat Commun.

[64] Bradley AJ, Green MJ (1997). Clinical mastitis in dairy cows after ‘blitz’ therapy. Vet Rec.

[65] Zaura E (2015). Same exposure but two radically different responses to antibiotics: resilience of the salivary microbiome versus long-term microbial shifts in feces. MBio.

[66] Cabrera-Rubio R (2012). The human milk microbiome changes over lactation and is shaped by maternal weight and mode of delivery. Am J Clin Nutr.

[67] Fitzstevens JL, et al. Systematic Review of the Human Milk Microbiota. Nutr Clin Pract. 2017;32(3):354–364. doi:10.1177/0884533616670150. 10.1177/0884533616670150 27679525

[68] Burvenich C (2003). Severity of E. coli mastitis is mainly determined by cow factors. Vet Res.

[69] Keane OM (2016). Genetic diversity, the virulence gene profile and antimicrobial resistance of clinical mastitis-associated Escherichia coli. Res Microbiol.

[70] Richards VP (2015). Genome based phylogeny and comparative genomic analysis of intra-mammary pathogenic Escherichia coli. PLoS One.

[71] Blagitz MG (2015). Flow cytometric analysis: Interdependence of healthy and infected udder quarters. J Dairy Sci.

[72] Merle R, Schroder A, Hamann J (2007). Cell function in the bovine mammary gland: a preliminary study on interdependence of healthy and infected udder quarters. J Dairy Res.

[73] Jensen K (2013). Escherichia coli- and Staphylococcus aureus-induced mastitis differentially modulate transcriptional responses in neighbouring uninfected bovine mammary gland quarters. BMC Genomics.

