# Supplementary material for: Normal milk microbiome is reestablished following experimental infection with Escherichia coli independent of intramammary antibiotic treatment with a third-generation cephalosporin in bovines
Source: Microbiome. 2017 Jul 12;5:74. doi: 10.1186/s40168-017-0291-5 (PMC5506599; doi:10.1186/s40168-017-0291-5)
Supplement: Supplementary file 1 — Detailed information on bacterial profile per study. animal (PDF 888 kb) [file 40168_2017_291_MOESM1_ESM.pdf]

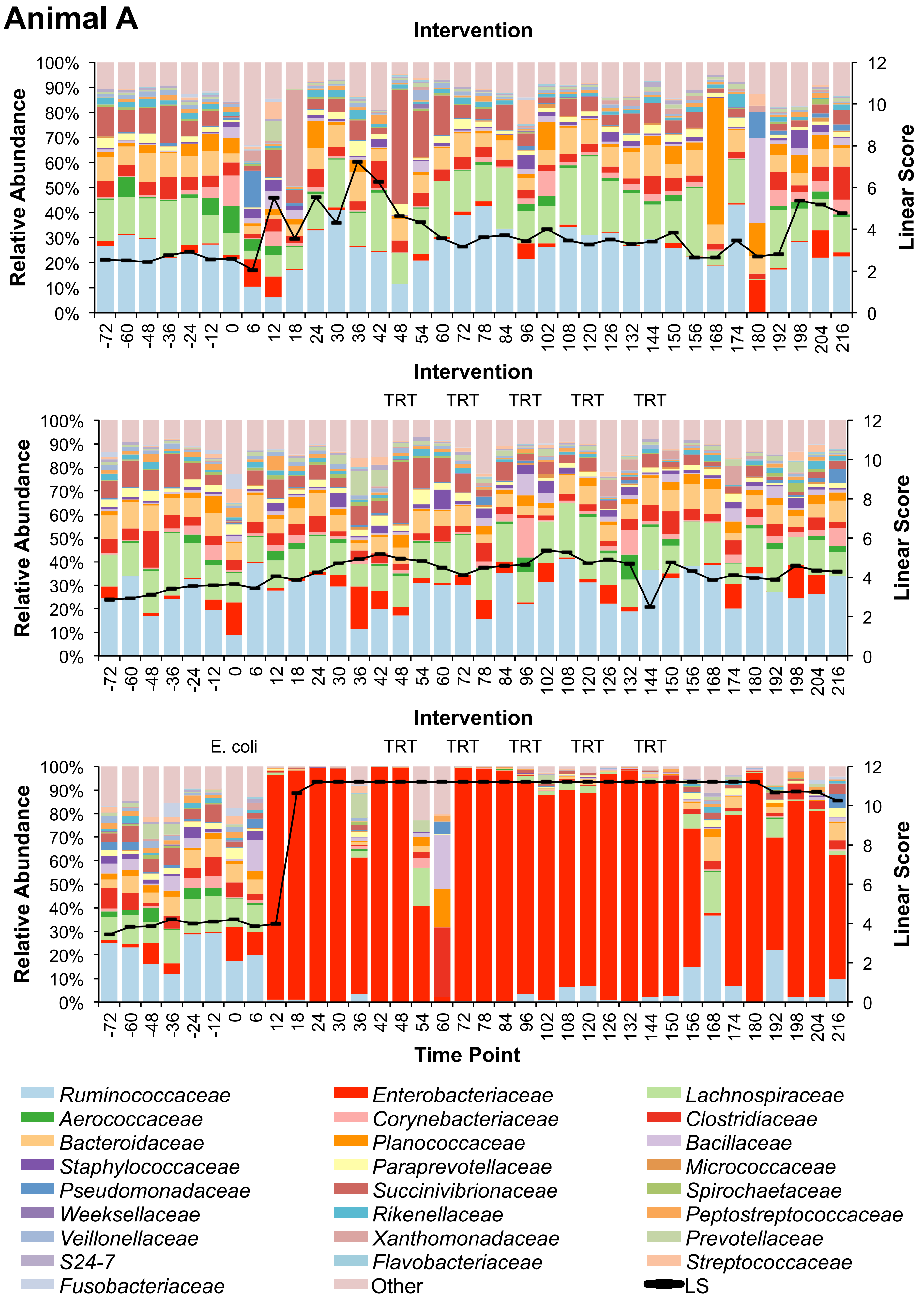

Animal A
Intervention
Intervention
TRT
TRT
TRT
TRT
TRT
Intervention
E. coli
TRT
TRT
TRT
TRT
TRT
Relative Abundance
Linear Score
Time Point
-72
-60
-48
-36
-24
-12
0
6
12
18
24
30
36
42
48
54
60
72
78
84
96
102
108
120
126
132
144
150
156
168
174
180
192
198
204
216
Ruminococcaceae
Enterobacteriaceae
Lachnospiraceae
Aerococcaceae
Corynebacteriaceae
Clostridiaceae
Bacteroidaceae
Planococcaceae
Bacillaceae
Staphylococcaceae
Paraprevotellaceae
Micrococcaceae
Pseudomonadaceae
Succinivibrionaceae
Spirochaetaceae
Weeksellaceae
Rikenellaceae
Peptostreptococcaceae
Veillonellaceae
Xanthomonadaceae
Prevotellaceae
S24-7
Flavobacteriaceae
Streptococcaceae
Fusobacteriaceae
Other
LS

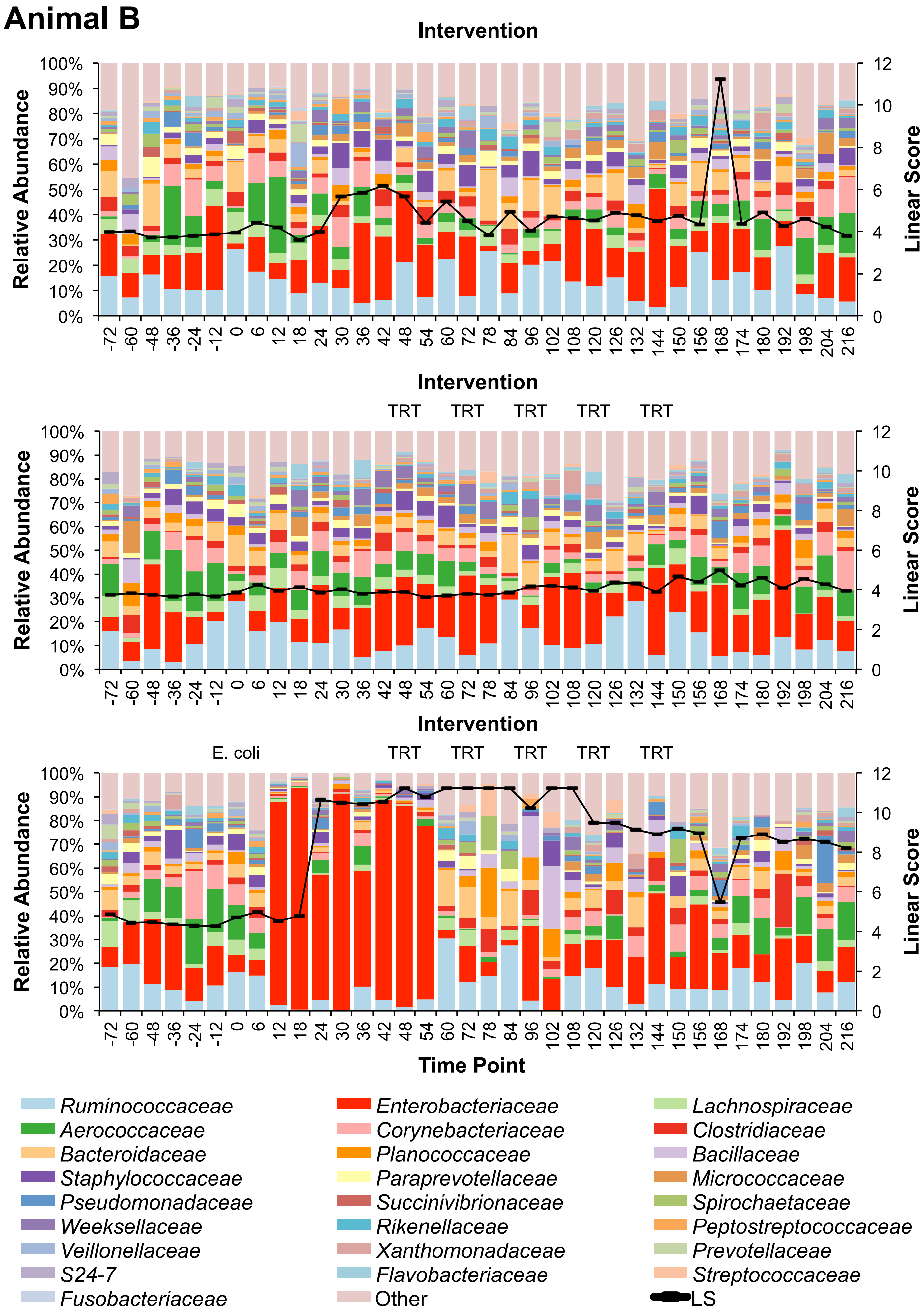

Animal B
Intervention
TRT TRT TRT TRT TRT
E. coli
Relative Abundance
Linear Score
Time Point
0% 10% 20% 30% 40% 50% 60% 70% 80% 90% 100%
0 2 4 6 8 10 12
-72 -60 -48 -36 -24 -12 0 6 12 18 24 30 36 42 48 54 60 72 78 84 96 102 108 120 126 132 144 150 156 168 174 180 192 198 204 216
Ruminococcaceae
Aerococcaceae
Bacteroidaceae
Staphylococcaceae
Pseudomonadaceae
Weeksellaceae
Veillonellaceae
S24-7
Fusobacteriaceae
Enterobacteriaceae
Corynebacteriaceae
Planococcaceae
Paraprevotellaceae
Succinivibrionaceae
Rikenellaceae
Xanthomonadaceae
Flavobacteriaceae
Other
Lachnospiraceae
Clostridiaceae
Bacillaceae
Micrococcaceae
Spirochaetaceae
Peptostreptococcaceae
Prevotellaceae
Streptococcaceae
LS

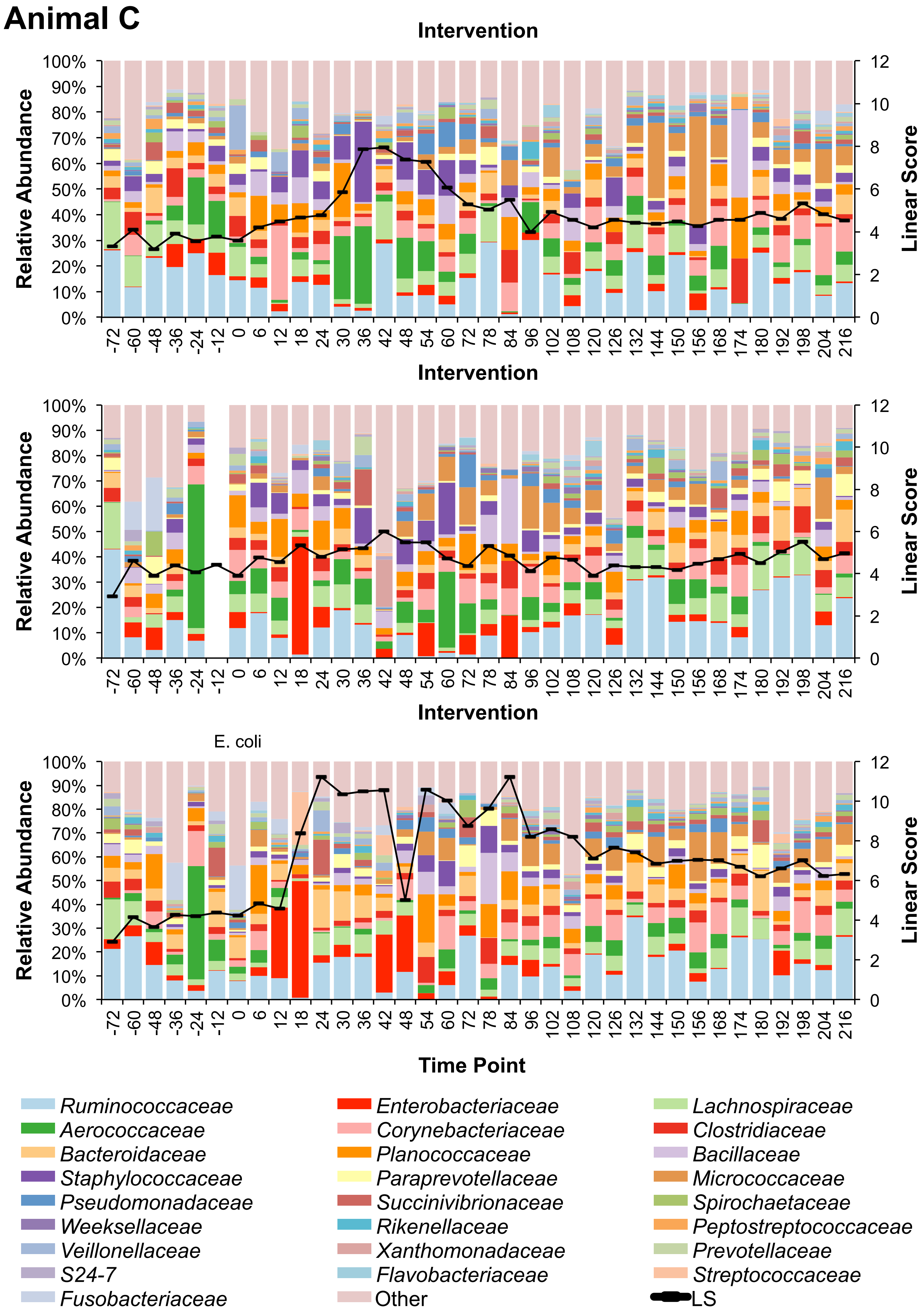

Animal C
Intervention
Relative Abundance
Linear Score
0%
10%
20%
30%
40%
50%
60%
70%
80%
90%
100%
0
2
4
6
8
10
12
-72
-60
-48
-36
-24
-12
0
6
12
18
24
30
36
42
48
54
60
72
78
84
96
102
108
120
126
132
144
150
156
168
174
180
192
198
204
216
E. coli
Time Point
Ruminococcaceae
Aerococcaceae
Bacteroidaceae
Staphylococcaceae
Pseudomonadaceae
Weeksellaceae
Veillonellaceae
S24-7
Fusobacteriaceae
Enterobacteriaceae
Corynebacteriaceae
Planococcaceae
Paraprevotellaceae
Succinivibrionaceae
Rikenellaceae
Xanthomonadaceae
Flavobacteriaceae
Other
Lachnospiraceae
Clostridiaceae
Bacillaceae
Micrococcaceae
Spirochaetaceae
Peptostreptococcaceae
Prevotellaceae
Streptococcaceae
LS

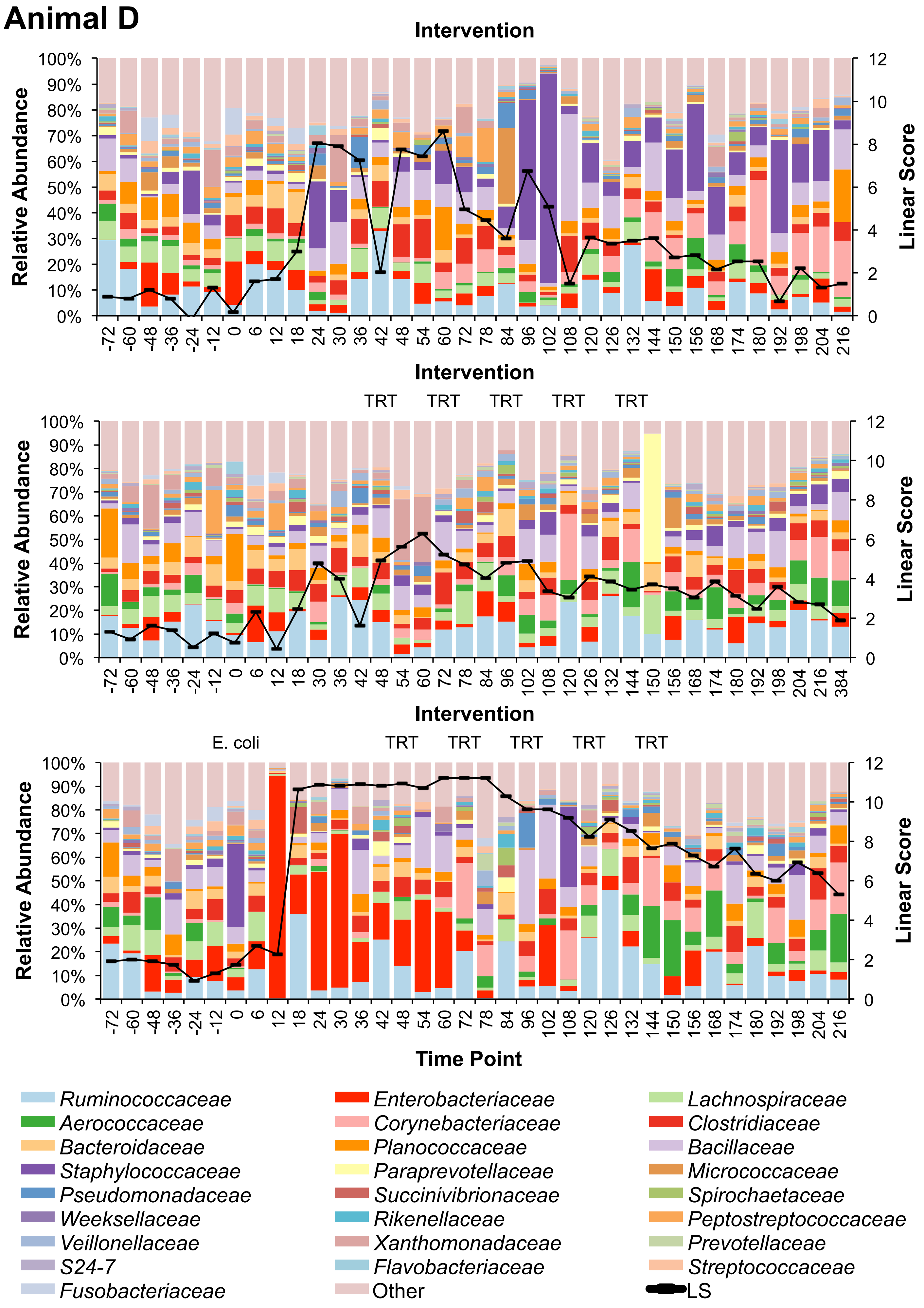
Animal D
Intervention
Intervention
TRT TRT TRT TRT TRT
Intervention
E. coli
TRT TRT TRT TRT TRT
Relative Abundance
Linear Score
100%
90%
80%
70%
60%
50%
40%
30%
20%
10%
0%
12
10
8
6
4
2
0
-72 -60 -48 -36 -24 -12 0 6 12 18 24 30 36 42 48 54 60 72 78 84 96 102 108 120 126 132 144 150 156 168 174 180 192 198 204 216
-72 -60 -48 -36 -24 -12 0 6 12 18 30 36 42 48 54 60 72 78 84 96 102 108 120 126 132 144 150 156 168 174 180 192 198 204 216 384
-72 -60 -48 -36 -24 -12 0 6 12 18 24 30 36 42 48 54 60 72 78 84 96 102 108 120 126 132 144 150 156 168 174 180 192 198 204 216
Time Point
Ruminococcaceae
Enterobacteriaceae
Lachnospiraceae
Aerococcaceae
Corynebacteriaceae
Clostridiaceae
Bacteroidaceae
Planococcaceae
Bacillaceae
Staphylococcaceae
Paraprevotellaceae
Micrococcaceae
Pseudomonadaceae
Succinivibrionaceae
Spirochaetaceae
Weeksellaceae
Rikenellaceae
Peptostreptococcaceae
Veillonellaceae
Xanthomonadaceae
Prevotellaceae
S24-7
Flavobacteriaceae
Streptococcaceae
Fusobacteriaceae
Other
LS

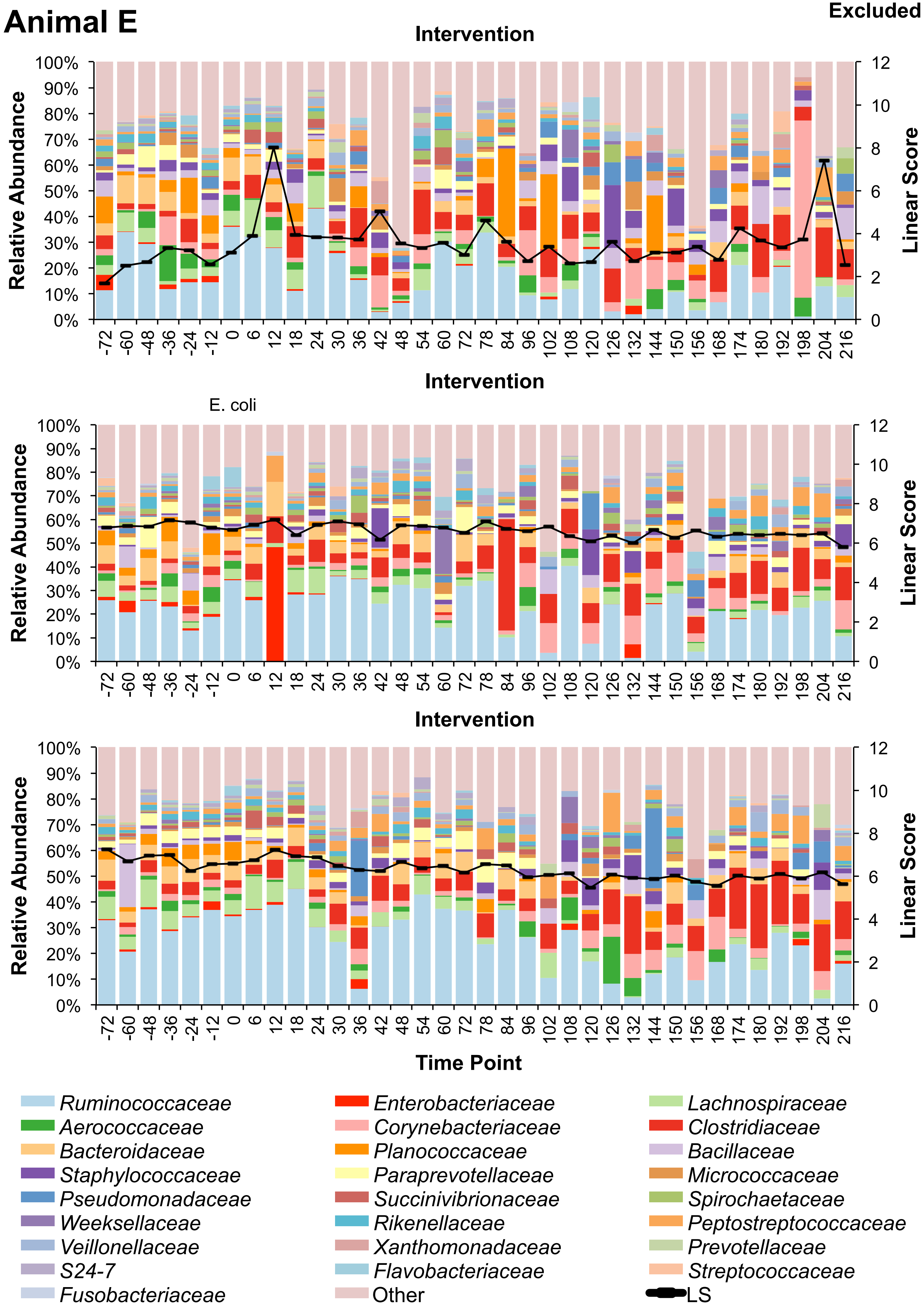

Animal E
Excluded
Intervention
E. coli
Relative Abundance
Linear Score
Time Point
0%
10%
20%
30%
40%
50%
60%
70%
80%
90%
100%
0
2
4
6
8
10
12
-72
-60
-48
-36
-24
-12
0
6
12
18
24
30
36
42
48
54
60
72
78
84
96
102
108
120
126
132
144
150
156
168
174
180
192
198
204
216
Ruminococcaceae
Enterobacteriaceae
Lachnospiraceae
Aerococcaceae
Corynebacteriaceae
Clostridiaceae
Bacteroidaceae
Planococcaceae
Bacillaceae
Staphylococcaceae
Paraprevotellaceae
Micrococcaceae
Pseudomonadaceae
Succinivibrionaceae
Spirochaetaceae
Weeksellaceae
Rikenellaceae
Peptostreptococcaceae
Veillonellaceae
Xanthomonadaceae
Prevotellaceae
S24-7
Flavobacteriaceae
Streptococcaceae
Fusobacteriaceae
Other
LS

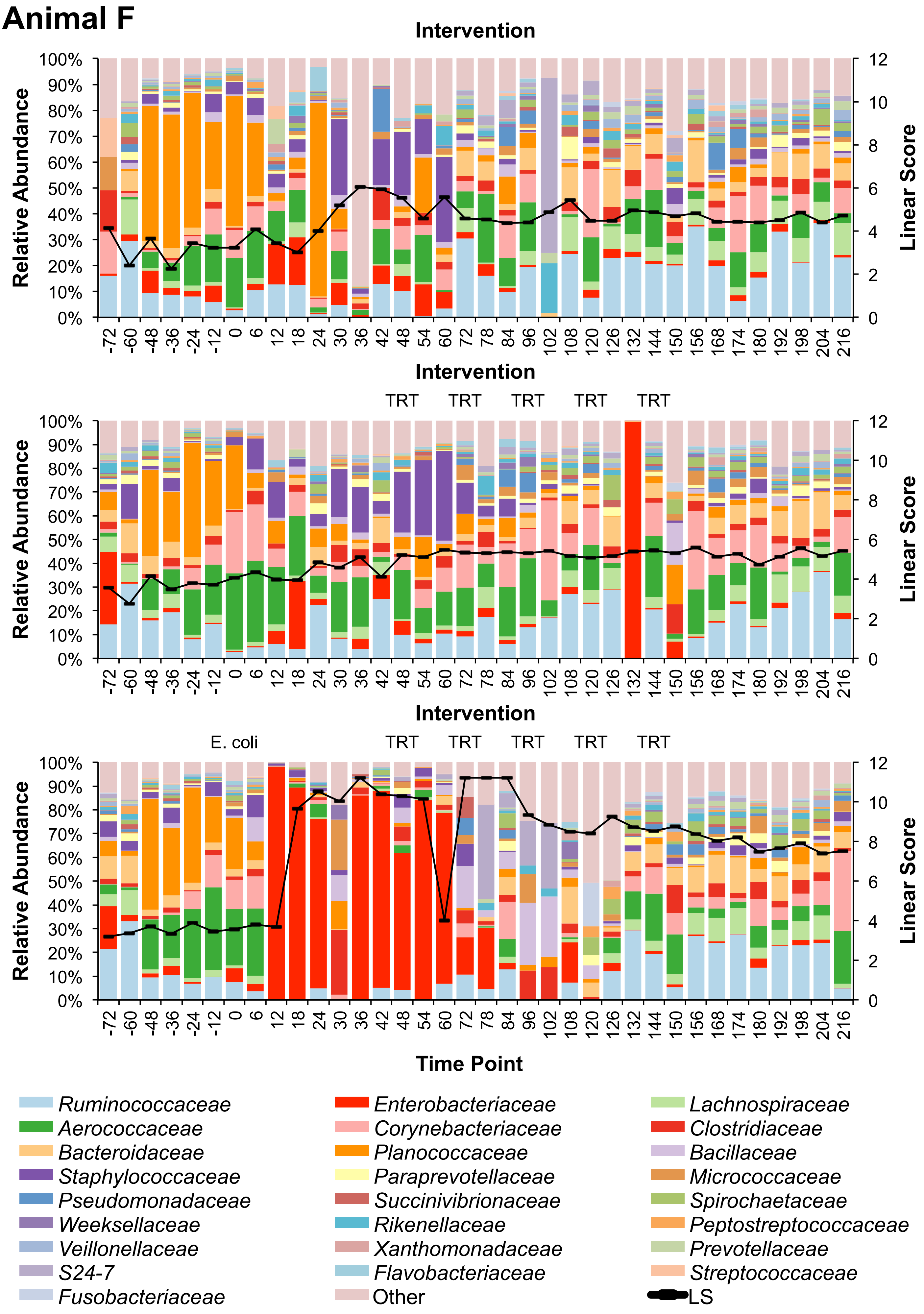
Animal F
Intervention
Relative Abundance
Linear Score
100%
90%
80%
70%
60%
50%
40%
30%
20%
10%
0%
12
10
8
6
4
2
0
-72 -60 -48 -36 -24 -12 0 6 12 18 24 30 36 42 48 54 60 72 78 84 96 102 108 120 126 132 144 150 156 168 174 180 192 198 204 216
Intervention
TRT TRT TRT TRT TRT
Intervention
E. coli
TRT TRT TRT TRT TRT
Time Point
Ruminococcaceae
Enterobacteriaceae
Lachnospiraceae
Aerococcaceae
Corynebacteriaceae
Clostridiaceae
Bacteroidaceae
Planococcaceae
Bacillaceae
Staphylococcaceae
Paraprevotellaceae
Micrococcaceae
Pseudomonadaceae
Succinivibrionaceae
Spirochaetaceae
Weeksellaceae
Rikenellaceae
Peptostreptococcaceae
Veillonellaceae
Xanthomonadaceae
Prevotellaceae
S24-7
Flavobacteriaceae
Streptococcaceae
Fusobacteriaceae
Other
LS

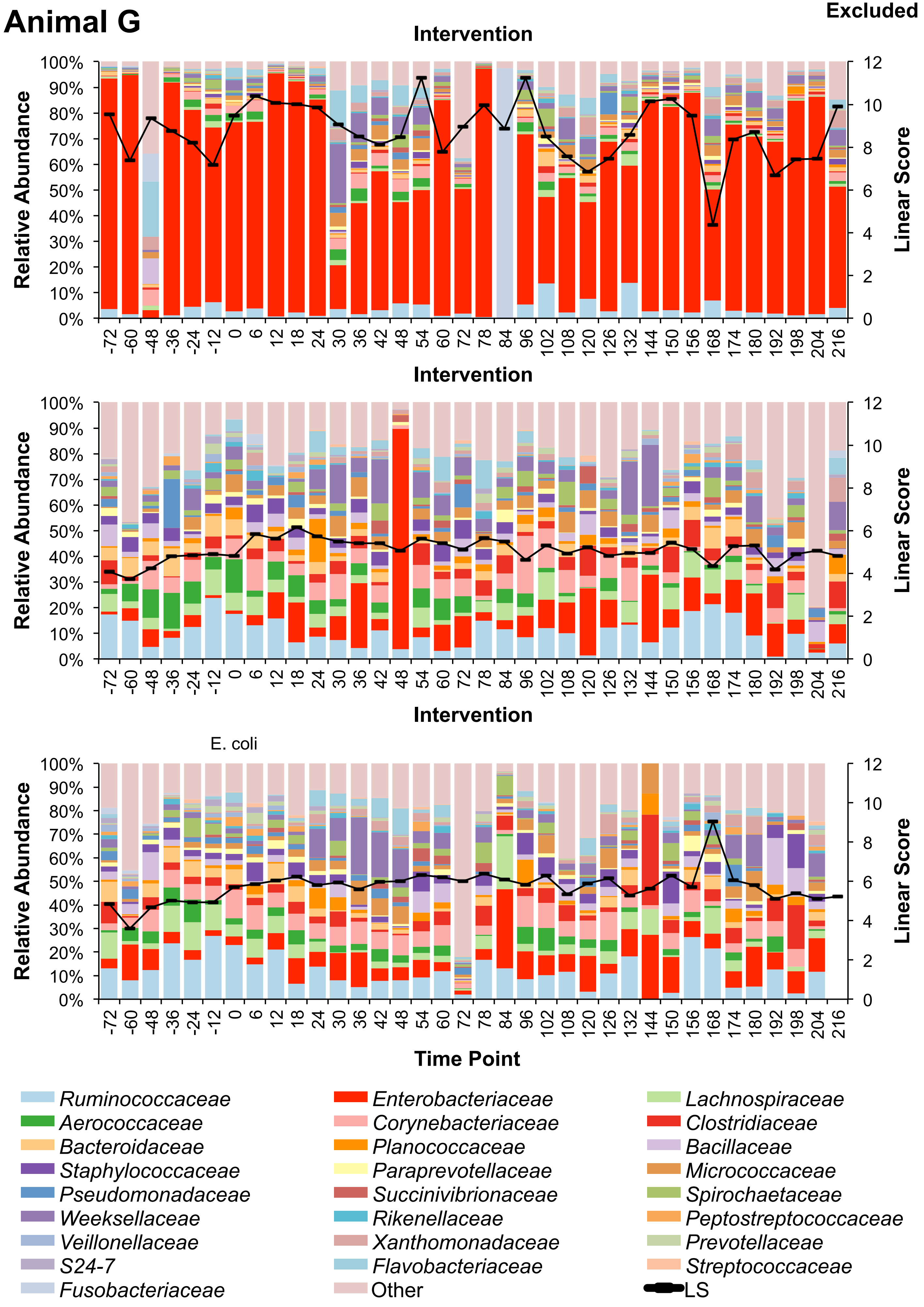

Animal G
Excluded
Intervention
Intervention
Intervention
E. coli
Relative Abundance
Linear Score
Time Point
Ruminococcaceae
Aerococcaceae
Bacteroidaceae
Staphylococcaceae
Pseudomonadaceae
Weeksellaceae
Veillonellaceae
S24-7
Fusobacteriaceae
Enterobacteriaceae
Corynebacteriaceae
Planococcaceae
Paraprevotellaceae
Succinivibrionaceae
Rikenellaceae
Xanthomonadaceae
Flavobacteriaceae
Other
Lachnospiraceae
Clostridiaceae
Bacillaceae
Micrococcaceae
Spirochaetaceae
Peptostreptococcaceae
Prevotellaceae
Streptococcaceae
LS

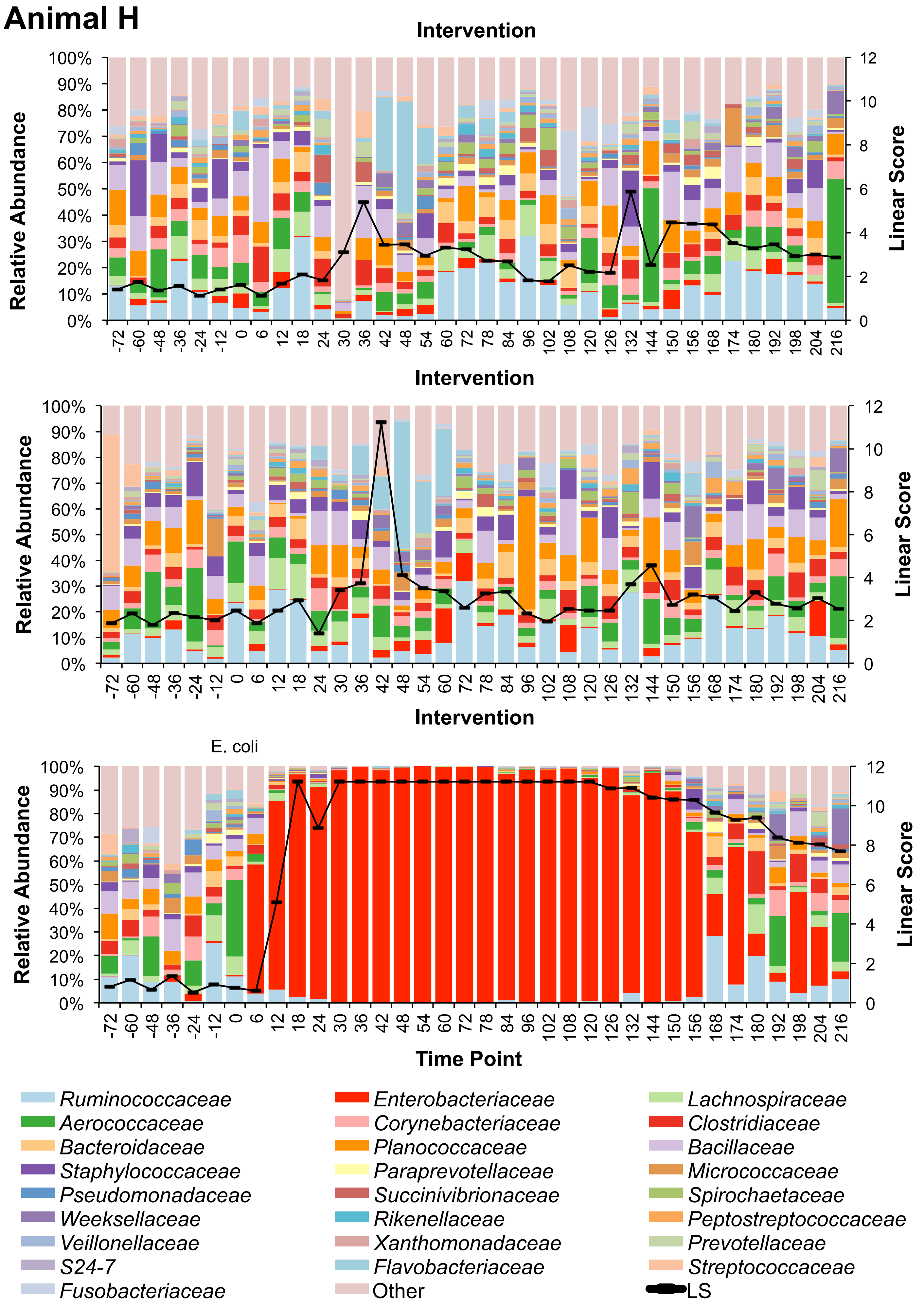

Animal H
Intervention
Relative Abundance
Linear Score
100%
90%
80%
70%
60%
50%
40%
30%
20%
10%
0%
12
10
8
6
4
2
0
-72 -60 -48 -36 -24 -12 0 6 12 18 24 30 36 42 48 54 60 72 78 84 96 102 108 120 126 132 144 150 156 168 174 180 192 198 204 216
Intervention
E. coli
Time Point
Ruminococcaceae
Aerococcaceae
Bacteroidaceae
Staphylococcaceae
Pseudomonadaceae
Weeksellaceae
Veillonellaceae
S24-7
Fusobacteriaceae
Enterobacteriaceae
Corynebacteriaceae
Planococcaceae
Paraprevotellaceae
Succinivibrionaceae
Rikenellaceae
Xanthomonadaceae
Flavobacteriaceae
Other
Lachnospiraceae
Clostridiaceae
Bacillaceae
Micrococcaceae
Spirochaetaceae
Peptostreptococcaceae
Prevotellaceae
Streptococcaceae
LS

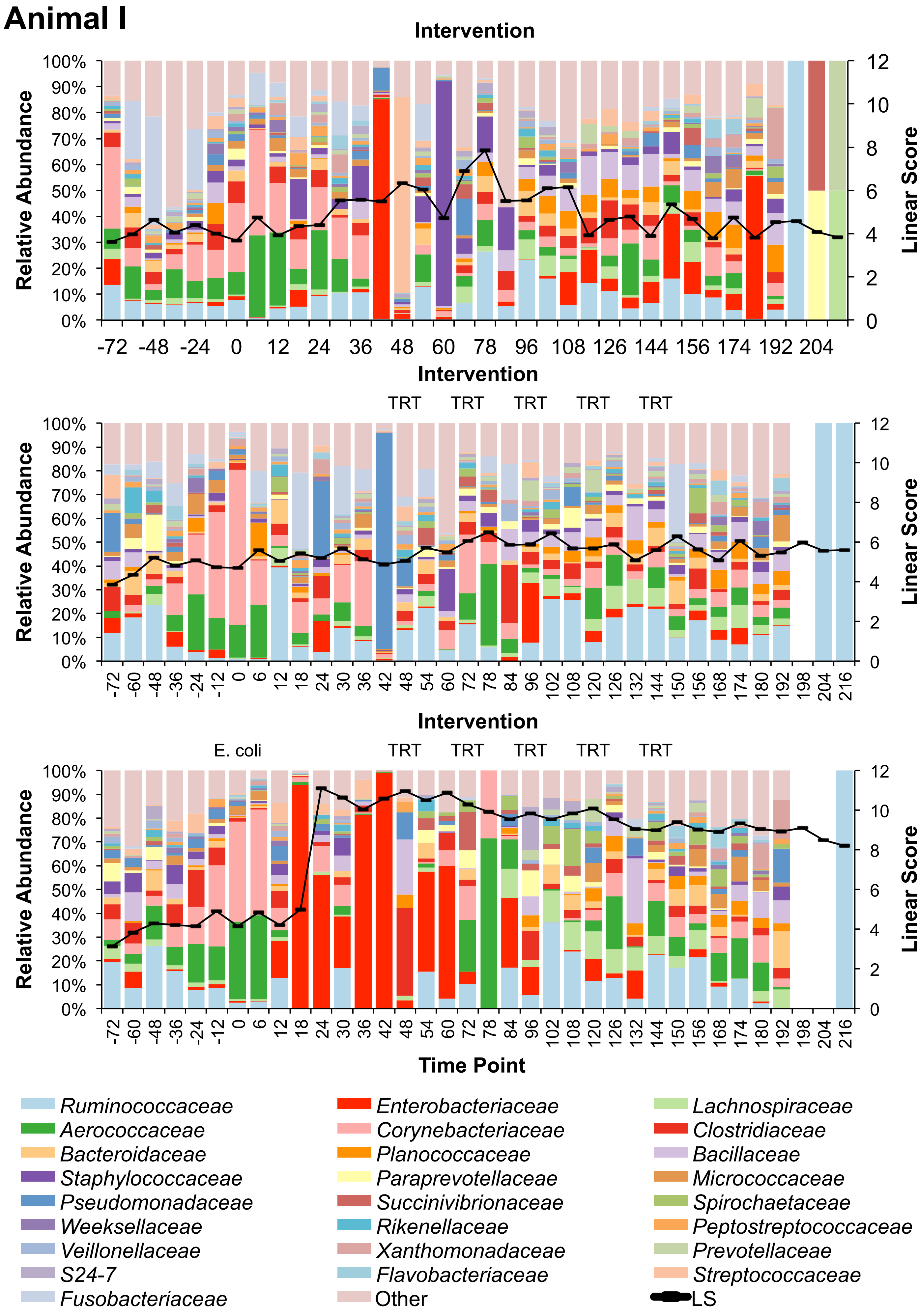
Animal I
Intervention
Relative Abundance
Linear Score
100% 90% 80% 70% 60% 50% 40% 30% 20% 10% 0%
12 10 8 6 4 2 0
-72 -48 -24 0 12 24 36 48 60 78 96 108 126 144 156 174 192 204
Intervention
TRT TRT TRT TRT TRT
Relative Abundance
Linear Score
-72 -60 -48 -36 -24 -12 0 6 12 18 24 30 36 42 48 54 60 72 78 84 96 102 108 120 126 132 144 150 156 168 174 180 192 198 204 216
Intervention
E. coli
TRT TRT TRT TRT TRT
Relative Abundance
Linear Score
-72 -60 -48 -36 -24 -12 0 6 12 18 24 30 36 42 48 54 60 72 78 84 96 102 108 120 126 132 144 150 156 168 174 180 192 198 204 216
Time Point
Ruminococcaceae
Aerococcaceae
Bacteroidaceae
Staphylococcaceae
Pseudomonadaceae
Weeksellaceae
Veillonellaceae
S24-7
Fusobacteriaceae
Enterobacteriaceae
Corynebacteriaceae
Planococcaceae
Paraprevotellaceae
Succinivibrionaceae
Rikenellaceae
Xanthomonadaceae
Flavobacteriaceae
Other
Lachnospiraceae
Clostridiaceae
Bacillaceae
Micrococcaceae
Spirochaetaceae
Peptostreptococcaceae
Prevotellaceae
Streptococcaceae
LS

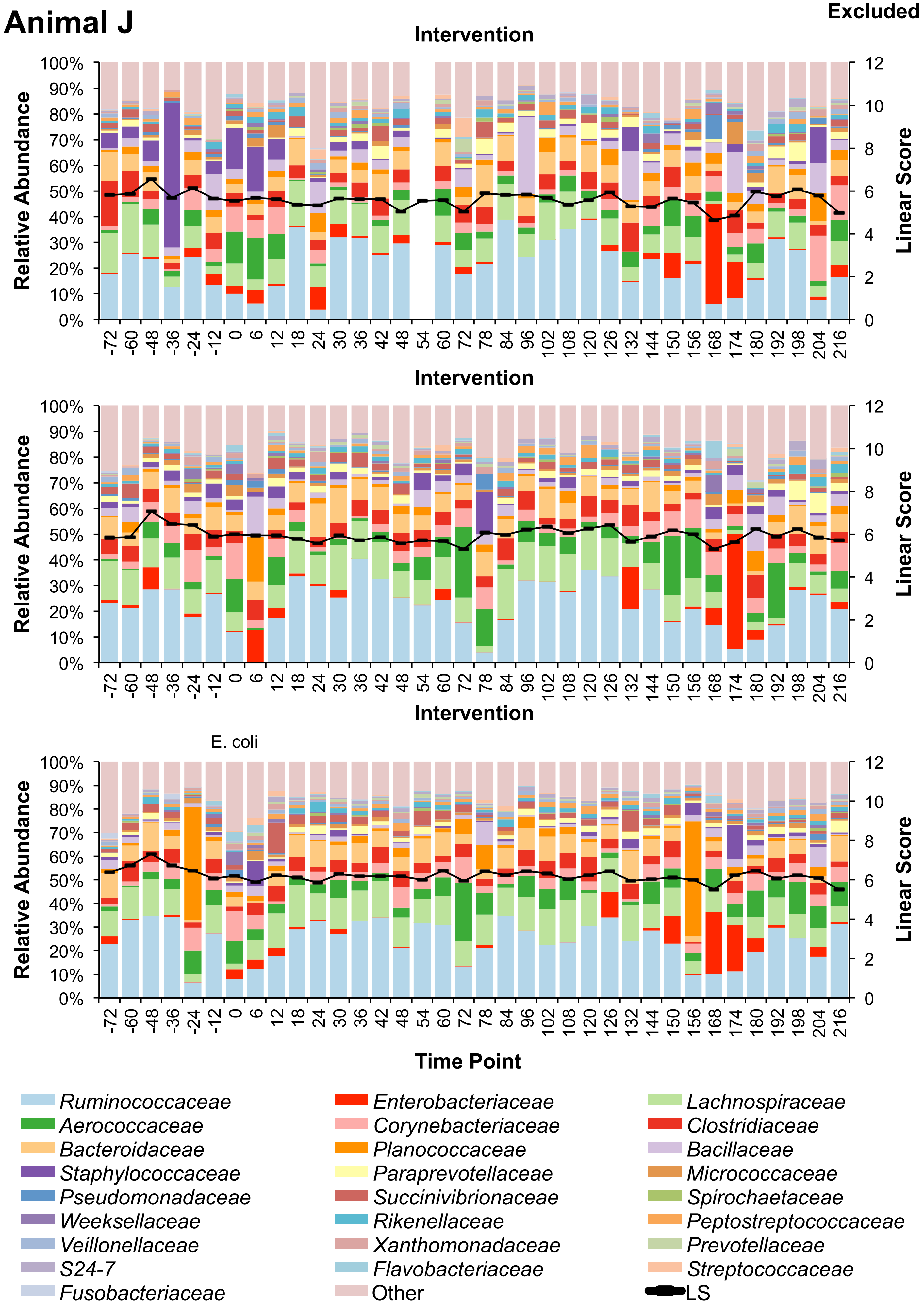
Animal J
Excluded
Intervention
E. coli
Relative Abundance
Linear Score
Time Point
100%
90%
80%
70%
60%
50%
40%
30%
20%
10%
0%
12
10
8
6
4
2
0
-72
-60
-48
-36
-24
-12
0
6
12
18
24
30
36
42
48
54
60
72
78
84
96
102
108
120
126
132
144
150
156
168
174
180
192
198
204
216
Ruminococcaceae
Enterobacteriaceae
Lachnospiraceae
Aerococcaceae
Corynebacteriaceae
Clostridiaceae
Bacteroidaceae
Planococcaceae
Bacillaceae
Staphylococcaceae
Paraprevotellaceae
Micrococcaceae
Pseudomonadaceae
Succinivibrionaceae
Spirochaetaceae
Weeksellaceae
Rikenellaceae
Peptostreptococcaceae
Veillonellaceae
Xanthomonadaceae
Prevotellaceae
S24-7
Flavobacteriaceae
Streptococcaceae
Fusobacteriaceae
Other
LS

Animal K

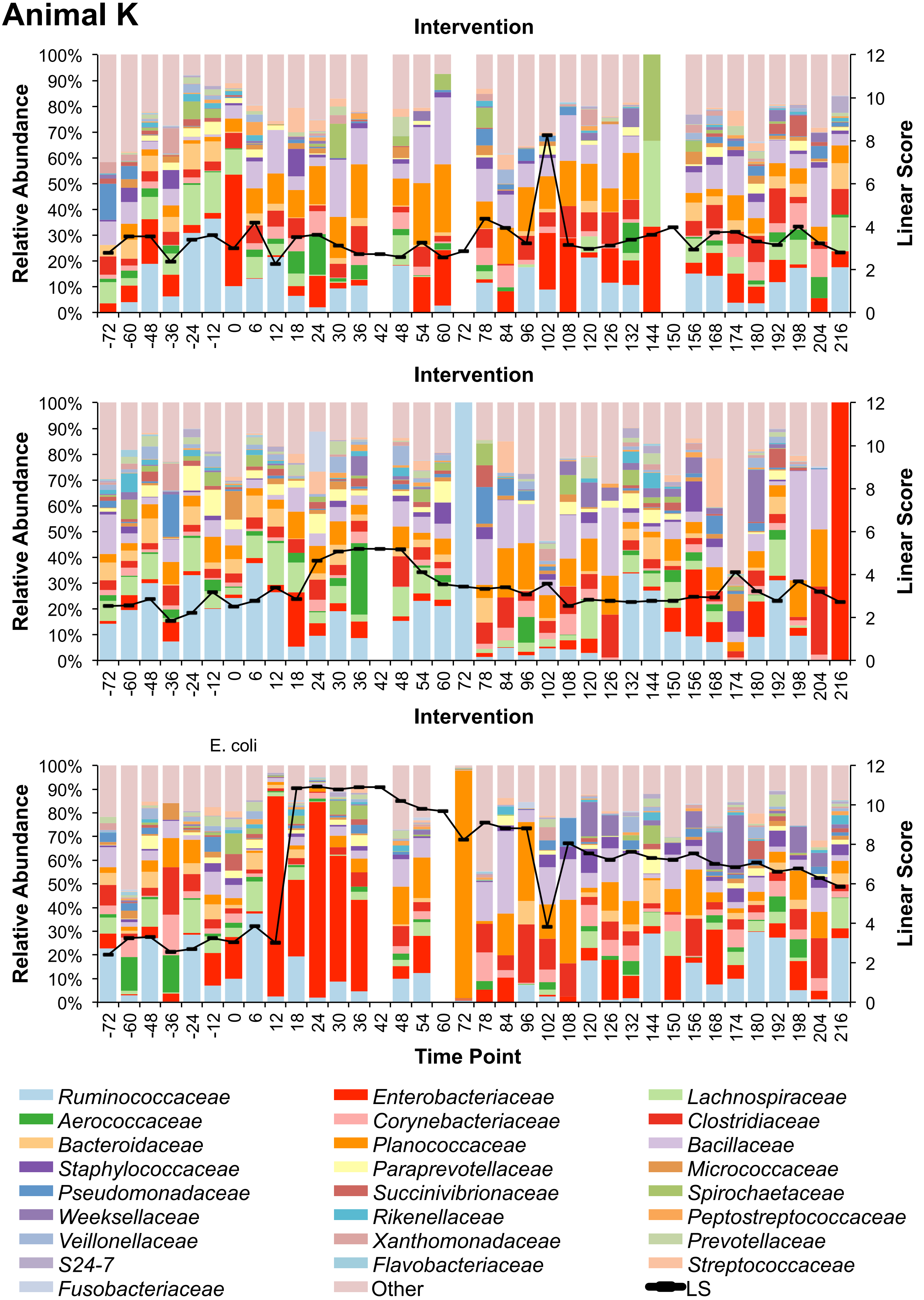
Intervention
Relative Abundance
Linear Score
100%
90%
80%
70%
60%
50%
40%
30%
20%
10%
0%
12
10
8
6
4
2
0
-72 -60 -48 -36 -24 -12 0 6 12 18 24 30 36 42 48 54 60 72 78 84 96 102 108 120 126 132 144 150 156 168 174 180 192 198 204 216
Intervention
Intervention
E. coli
Time Point
Ruminococcaceae
Enterobacteriaceae
Lachnospiraceae
Aerococcaceae
Corynebacteriaceae
Clostridiaceae
Bacteroidaceae
Planococcaceae
Bacillaceae
Staphylococcaceae
Paraprevotellaceae
Micrococcaceae
Pseudomonadaceae
Succinivibrionaceae
Spirochaetaceae
Weeksellaceae
Rikenellaceae
Peptostreptococcaceae
Veillonellaceae
Xanthomonadaceae
Prevotellaceae
S24-7
Flavobacteriaceae
Streptococcaceae
Fusobacteriaceae
Other
LS

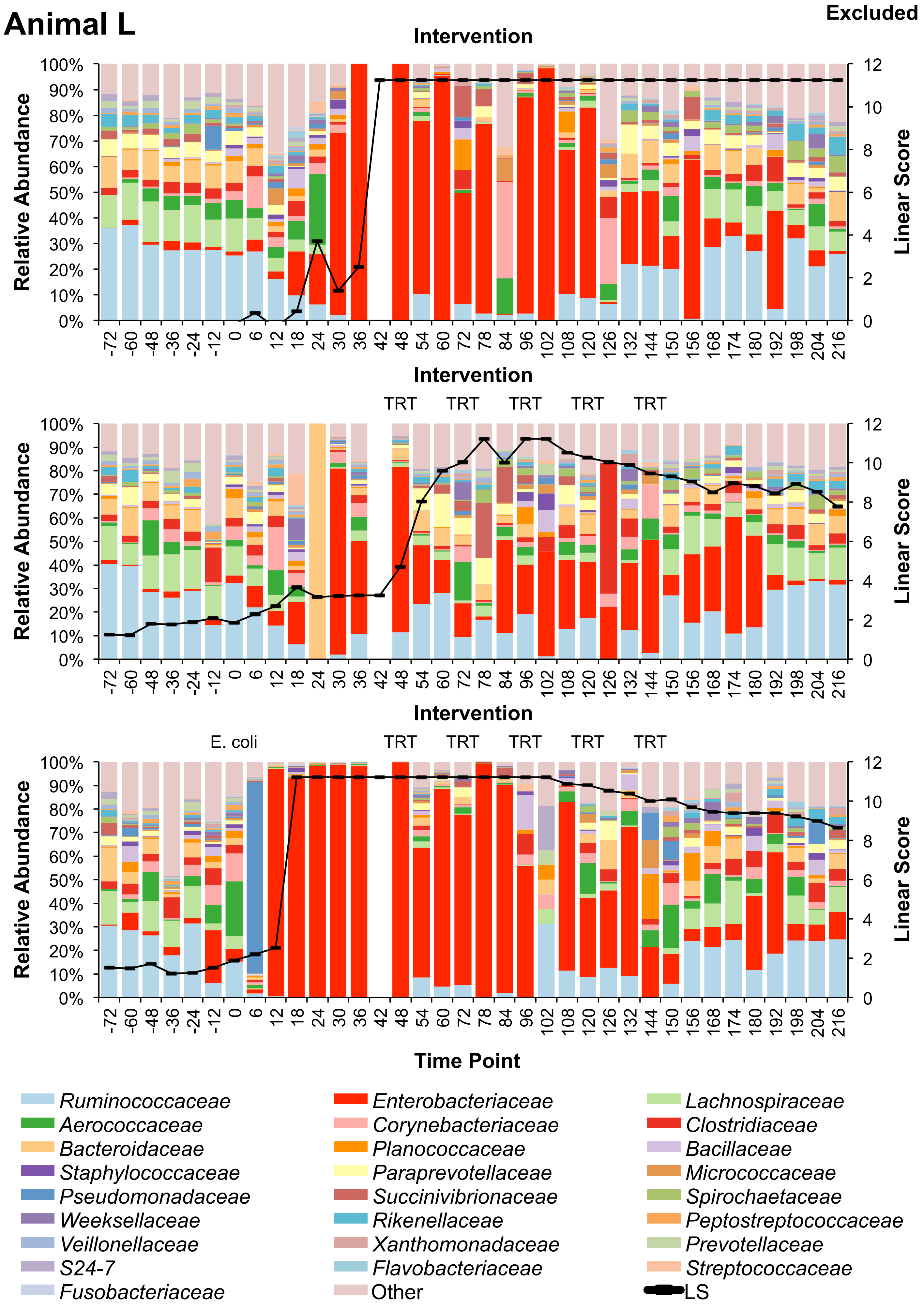

Animal L
Excluded
Intervention
Relative Abundance
Linear Score
100%
90%
80%
70%
60%
50%
40%
30%
20%
10%
0%
12
10
8
6
4
2
0
-72 -60 -48 -36 -24 -12 0 6 12 18 24 30 36 42 48 54 60 72 78 84 96 102 108 120 126 132 144 150 156 168 174 180 192 198 204 216
Intervention
TRT TRT TRT TRT TRT
Intervention
E. coli
TRT TRT TRT TRT TRT
Time Point
Ruminococcaceae
Enterobacteriaceae
Lachnospiraceae
Aerococcaceae
Corynebacteriaceae
Clostridiaceae
Bacteroidaceae
Planococcaceae
Bacillaceae
Staphylococcaceae
Paraprevotellaceae
Micrococcaceae
Pseudomonadaceae
Succinivibrionaceae
Spirochaetaceae
Weeksellaceae
Rikenellaceae
Peptostreptococcaceae
Veillonellaceae
Xanthomonadaceae
Prevotellaceae
S24-7
Flavobacteriaceae
Streptococcaceae
Fusobacteriaceae
Other
LS
